# Lipid mediators and biomarkers associated with type 1 diabetes development

**DOI:** 10.1172/jci.insight.138034

**Published:** 2020-08-20

**Authors:** Alexander J. Nelson, Daniel J. Stephenson, Robert N. Bone, Christopher L. Cardona, Margaret A. Park, Ying G. Tusing, Xiaoyong Lei, George Kokotos, Christina L. Graves, Clayton E. Mathews, Joanna Kramer, Martin J. Hessner, Charles E. Chalfant, Sasanka Ramanadham

**Affiliations:** 1 Department of Cell, Developmental, and Integrative Biology, and; 2 Comprehensive Diabetes Center, University of Alabama at Birmingham (UAB), Birmingham, Alabama, USA.; 3 Department of Cell Biology, Microbiology and Molecular Biology (CMMB), University of South Florida, Tampa, Florida, USA.; 4 Department of Medicine, Indiana University School of Medicine, Indianapolis, Indiana, USA.; 5 Laboratory of Organic Chemistry, Department of Chemistry, National and Kapodistrian University of Athens, Panepistimiopolis, Athens, Greece.; 6 Department of Biology, University of North Carolina, Chapel Hill, North Carolina, USA.; 7 Department of Pathology, Immunology, and Laboratory Medicine, College of Medicine, University of Florida Health Science Center, Gainesville, Florida, USA.; 8 Max McGee Research Center for Juvenile Diabetes, Department of Pediatrics at Medical College of Wisconsin and Children’s Research Institute of Children’s Hospital of Wisconsin, Milwaukee, Wisconsin, USA.; 9 Research Service, James A. Haley Veterans Hospital, Tampa, Florida, USA.

**Keywords:** Endocrinology, Inflammation, Autoimmune diseases, Diabetes, Macrophages

## Abstract

Type 1 diabetes (T1D) is a consequence of autoimmune β cell destruction, but the role of lipids in this process is unknown. We previously reported that activation of Ca^2+^-independent phospholipase A_2_β (iPLA_2_β) modulates polarization of macrophages (MΦ). Hydrolysis of the *sn*-2 substituent of glycerophospholipids by iPLA_2_β can lead to the generation of oxidized lipids (eicosanoids), pro- and antiinflammatory, which can initiate and amplify immune responses triggering β cell death. As MΦ are early triggers of immune responses in islets, we examined the impact of iPLA_2_β-derived lipids (iDLs) in spontaneous-T1D prone nonobese diabetic mice (NOD), in the context of MΦ production and plasma abundances of eicosanoids and sphingolipids. We find that (a) MΦ_NOD_ exhibit a proinflammatory lipid landscape during the prediabetic phase; (b) early inhibition or genetic reduction of iPLA_2_β reduces production of select proinflammatory lipids, promotes antiinflammatory MΦ phenotype, and reduces T1D incidence; (c) such lipid changes are reflected in NOD plasma during the prediabetic phase and at T1D onset; and (d) importantly, similar lipid signatures are evidenced in plasma of human subjects at high risk for developing T1D. These findings suggest that iDLs contribute to T1D onset and identify select lipids that could be targeted for therapeutics and, in conjunction with autoantibodies, serve as early biomarkers of pre-T1D.

## Abstract 2

Lipid signaling contributes to type 1 diabetes onset and may provide therapeutic targets and biomarkers for disease.

## Graphical abstract

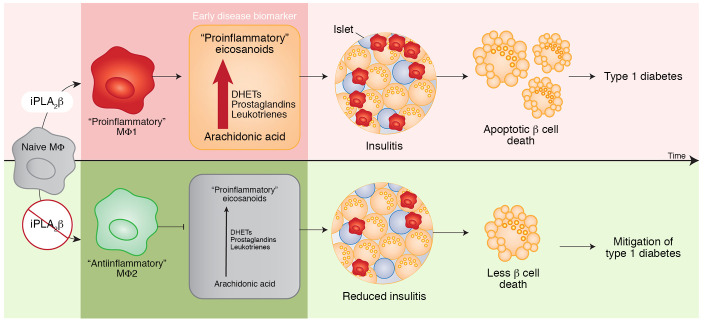

## Introduction

Type 1 diabetes (T1D) is a consequence of autoimmune destruction of β cells, involving activation of cellular immunity and inflammation initiated by early-stage immune cell infiltration of islets (1). While the roles of various stressors (i.e., cytokines, ROS, glucose) in this process have been studied extensively, the impact of lipids on β cell health during T1D development has not received significant attention. As such, there exists a significant gap in the understanding of how lipids generated by immune cells and/or β cells contribute to β cell demise.

Phospholipases A_2_ (PLA_2_s) hydrolyze the *sn*-2 substituent of glycerophospholipids to release a lysophospholipid and a free fatty acid (2). When the fatty acid is arachidonic acid, it can be metabolized by cyclooxygenases (COX), lipoxygenases (LOX), and cytochrome P450 (CYP) enzymes to generate oxidized bioactive lipids, or eicosanoids, which manifest a variety of effects. Some of the most potent inflammatory eicosanoids (3) are prostaglandin E_2_ (PGE_2_), leukotrienes (LTs), HETEs, and dihydroxyeicosatrienoic acids (DHETs), and they contribute to autoimmune diseases (4).

Among the PLA_2_s is a Ca^2+^-independent phospholipase A_2_ (iPLA_2_β), and its activity promotes deleterious outcomes in experimental and clinical diabetes (5, 6). Immune cells express iPLA_2_β (7), and inhibition of iPLA_2_β reduces generation of ROS (7), as well as antibody production from B cells and TNF-α from CD4^+^ T cells (8) and macrophages (MΦ) (9). Inhibition of iPLA_2_β has been shown to be effective in countering autoimmunity (10) and inflammation (11). Islet-resident MΦ and early islet-infiltrating MΦ promote infiltration of other immune cells, with M1 proinflammatory MΦ (12) recognized as causative factors in T1D development (13), whereas M2 antiinflammatory MΦ (14) are protective against T1D (15).

We and others have demonstrated that iPLA_2_β participates in β cell apoptosis (16, 17) and modulates MΦ polarization (18, 19). In light of these observations, we herein used lipidomics to gain insight into the lipidome associated with T1D development in NOD mice (hereafter referred to as NOD) and humans at high risk for developing T1D.

## Results

### Nomenclature

Mice used in these studies included spontaneous diabetes–resistant C57BL/6J, spontaneous diabetes–prone NOD, and NOD.*PLA2G6*^+/–^. These strains are designated C57, NOD, and NOD-HET, respectively. MΦ from these are designated MΦ_C57_, MΦ_NOD_, and MΦ_NOD-HET_, respectively.

### Age-dependent impact of iPLA_2_β inhibition on T1D development

**Figure 1:**
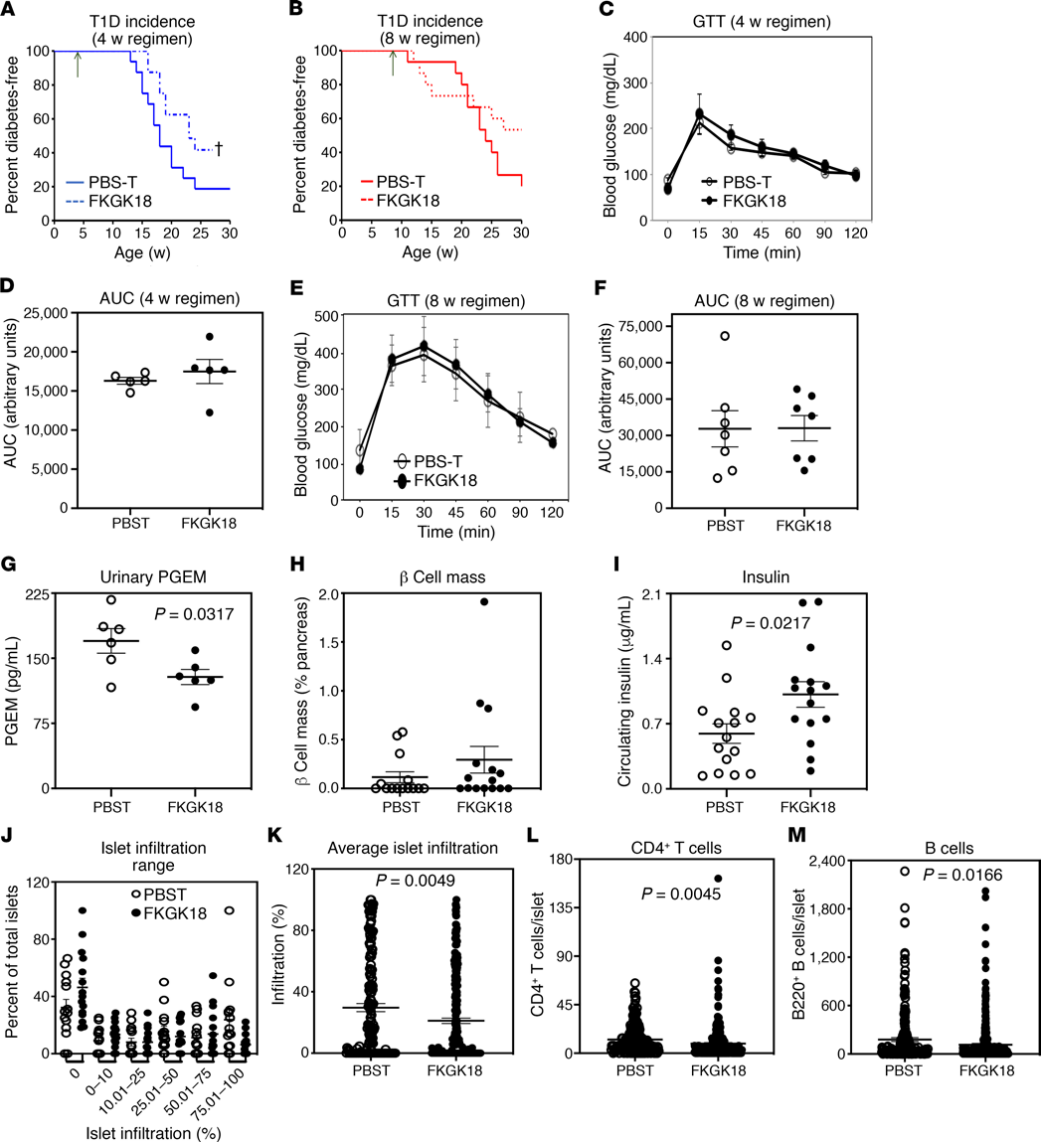
Effects of temporal FKGK18 regimen on T1D incidence and islet phenotype. Female NOD mice were administered FKGK18 (20 mg/kg, 3 × weekly) or vehicle (PBS-T) starting at 4 or 8 weeks of age. (**A** and **B**) Diabetes incidence. Blood glucose was monitored weekly in the 4-week (**A**; *n* = 17 and 15 for PBS-T and FKGK18 groups, respectively) and 8-week (**B**; *n* = 15 each in the PBS-T and FKGK18 groups) regimen groups for up to 30 weeks. Two consecutive readings of ≥ 275 mg/dL were recorded as onset of T1D (^†^*P* < 0.05). (**C–F**) Glucose tolerance test (GTT). Overnight fasted mice were administered glucose (2 g/kg, i.p.), glucose levels in blood from tail vein were monitored over a 2-hour period, and AUC were generated. (**C** and **D**) Four-week group at 14 weeks of age; *n* = 5 each in the PBS-T and FKGK18 groups. (**E** and **F**) Eight-week group at 25 weeks of age; *n* = 7 and 5 for PBS-T and FKGK18 groups, respectively. (**G–I**) Phenotype parameters in the 8-week regimen group. (**G**) Urinary PGE_2_ metabolites (PGEMs, *n* = 6 in each group, 18 weeks of age). (**H** and **I**) β Cell mass (PBS-T, *n* = 15; FKGK18, *n* = 14) (**H**) and circulating insulin (*n* = 15 in each group) (**I**) were determined at sacrifice (PBS-T, 14–30 weeks of age; FKGK18, 16–36 weeks of age). (**J** and **K**) Islet infiltration. Paraffin sections (10 μm) of pancreas were prepared and stained with H&E. Percent infiltration for each islet was calculated as the value of noninfiltrated area subtracted from total islet area (% infiltrate = 100 × [(total area – noninfiltrated area)/(total area)]) using ImageJ software. (PBS-T, *n* = 14 and 166 islets; FKGK18, *n* = 15 and 260 islets). (**J**) Islet Infiltration Range. (**K**) Average islet infiltration. (**L** and **M**) Islet immune cell phenotype. Paraffin sections (10 μm) of pancreas were prepared and stained for CD4^+^-T cells or B (B220) cells. Data presented are mean ± SEM of CD4^+^ T cells or B cells per islet. (**L**) Quantitation of CD4^±^ T cells per islet (PBS-T, *n* = 14 and 223 islets; FKGK18, *n* = 15 and 290 islets). (**M**) Quantitation of B cells per islet (PBS-T, *n* = 14 and 213 islets; FKGK18, *n* = 15 and 328 islets). Statistical analyses: (**A** and **B**) Mantel-Cox test; (**D–M**) Student’s *t* test.

As the female NOD exhibit a recognized progression in T1D development (1), where onset of insulitis commences at about 4 weeks of age and the inflammatory processes ramp up at about 8 weeks of age, we monitored T1D development in female NOD administered FKGK18 starting at 10 days and 4 or 8 weeks of age. Similar to our earlier report (8), 80%–90% of vehicle-treated NOD became diabetic by 25–30 weeks of age in the 10-day group, but only 10%–15% NOD administered FKGK18 developed T1D (data not shown). The vehicle-treated (PBS-T–treated) groups in the 4-week (Figure 1A) and 8-week (Figure 1B) groups also exhibited an 80% T1D incidence by 25–30 weeks of age. In contrast, 40% of mice in the 4-week FKGK18 group remained diabetes free (Figure 1A). While there was evidence of a modest delay in T1D incidence in the 8-week FKGK18 group (Figure 1B), it was not significantly different from the corresponding PBS-T group. No differences in glucose tolerance were noted between the groups started on PBS-T and FKGK18 at either 4 weeks (Figure 1, C and D) or 8 weeks of age (Figure 1, E and F).

Because the 8-week FKGK18-treated group appeared to be at the cusp of effective iPLA_2_β intervention, we further probed β cell and islet immune cell phenotype in this group. As expected, FKGK18 administration reduced urinary PGE_2_ metabolites (PGEM, Figure 1G), relative to the vehicle-treated mice, reflecting in vivo FKGK18-mediated inhibition of iPLA_2_β activity. This was accompanied by similar β cell mass (Figure 1H), higher circulating insulin (Figure 1I), and reduced islet infiltration (Figure 1, J and K). Furthermore, pancreatic islet abundances of CD4^+^ T cells (Figure 1L) and B cells (Figure 1M) were significantly reduced in the FKGK18-treated mice, relative to the PBS-T group. These findings reveal an age-dependent impact of iPLA_2_β inhibition on T1D, with early intervention being more beneficial.

### Protective effects of iPLA_2_β inhibition are lost upon FKGK18 withdrawal

**Figure 2:**
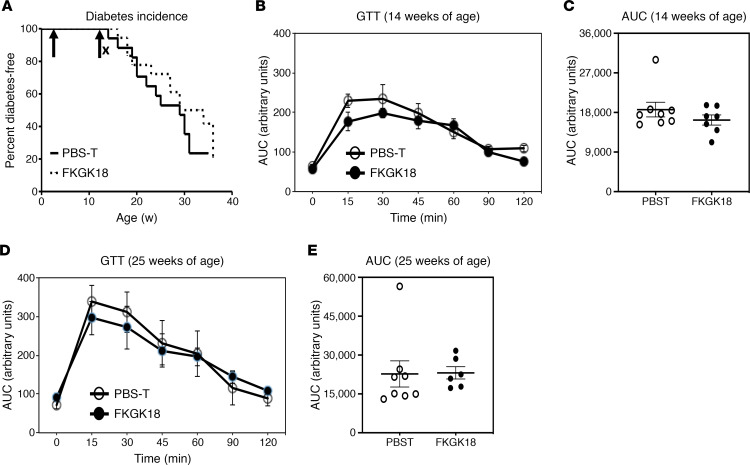
Effects of FKGK18-withdrawal regimen on T1D incidence and glucose tolerance. Female NOD mice were administered FKGK18 (20 mg/kg, 3 × weekly, *n* = 18) or vehicle (PBS-T, *n* = 17) starting at 10 days of age and until 14 weeks of age. (**A**) T1D incidence. Blood glucose was monitored weekly for up to 30 weeks, and 2 consecutive readings of ≥ 275 mg/dL were recorded as onset of T1D. (**B** and **D**) Glucose tolerance test (GTT). Assessed at 14 (**B**) and 25 (**D**) weeks of age (data are presented as mean ± SEM), as described in Figure 1. (**C** and **E**) Corresponding AUC. *N* values for PBS-T & FKGK18: 8 and 8 (**B** and **C**); , 8 and 6 (**D** and **E**), respectively. Statistical analyses: (**A**) Mantel-Cox test; (**C** and **E**) Student’s *t* test.

To determine if the protective effects of early intervention persist following inhibitor withdrawal, a concurrent cohort NOD group administered FKGK18 from 10 days until 14 weeks of age, an age closely associated with onset of T1D (20), was monitored for up to 30 weeks. We find that the decreased incidence in the NOD treated with FKGK18 continuously from 10 days until 30 weeks of age (8) was not evident when FKGK18 was withdrawn after 14 weeks (Figure 2A). Glucose tolerance was also indistinguishable between the PBS-T– and FKGK18-withdrawn groups (Figure 2, B–E). Taken together, these findings suggest that the protective effects of the reversible inhibitor FKGK18 are lost upon withdrawal.

### MΦ_NOD_ exhibit a profound inflammatory lipid profile

In view of the above observations suggesting a temporal impact of iPLA_2_β-derived lipids (iDL) on T1D development, we examined the lipid profile in the NOD, as compared with C57. MΦ are key to the autoimmune-mediated destruction of β cells, leading to T1D, as they are among the first cells to infiltrate the islets and trigger processes that promote infiltration of other immune cells (21, 22). Our earlier assessments of specific phenotypic markers revealed that iPLA_2_β activation promotes MΦ polarization toward M1, whereas iPLA_2_β deficiency favors M2 antiinflammatory polarization (18). We therefore targeted the MΦ lipid profile for analyses in the studies here. Peritoneal MΦ were isolated from mice and treated with either vehicle control (DMSO) or activated with IFN-γ + LPS. The media was collected for lipidomics analyses of eicosanoids, specialized proresolving mediators (SPMs), and fatty acids and the cells for sphingolipids. Multiple reaction monitoring (MRM) transitions with corresponding declustering potentials, collision energies, entrance potentials, and collision cell exit potentials are shown in Supplemental Tables 1 and 2 (supplemental material available online with this article; https://doi.org/10.1172/jci.insight.138034DS1).

#### Eicosanoids and fatty acids.

Metabolites of arachidonic acid are recognized to be pro- or antiinflammatory. In comparison with MΦ_C57_, production of several proinflammatory prostaglandins (PGs) by MΦ_NOD_ was significantly higher under both basal and activated conditions (Supplemental Table 3A). The most profoundly affected lipids included 6-keto PGF_1_α, 8-Iso PGF_2_α, 5-IPFα-VI, PGE_2_, PGA_2_, and 15-deoxy-Δ12,14-PGJ_2_. Furthermore, LT (LTD_4_, LTC_4_, and LTE_4_) production by MΦ_C57_ was significantly increased under basal conditions, and LTD_4_ production remained higher under activating conditions, in comparison with production by M_C57_. Production of HETEs, DHETs, or PGE_1_ was not significantly different between the 2 groups under basal conditions, but under activating conditions, production of 12-HETE, (±) 8,9-DHET, and PGE_1_ by MΦ_NOD_ was significantly higher, relative to MΦ_C57_ (Supplemental Table 3, A and B). Cellular lipidomic analyses identified several SPMs, including resolvin D2 and D1 (from docosahexaenoic acid [DHA]), and lipoxin A4 (from arachidonic acid [AA]). However, production of these or fatty acids EPA, DHA, and AA (Supplemental Table 3C) by MΦ_NOD_ and MΦ_C57_ was not different under basal or activated conditions.

#### Sphingolipids.

As our earlier studies revealed that stress-induced β cell death is associated with increases in various proapoptotic ceramides (CMs) (17, 23, 24), we assessed sphingolipids production by MΦ_NOD_. We found that several CM species (C16:0, C22:0, C24:1, C24:0) are higher in MΦ_NOD_ under both basal and classical activation, relative to MΦ_C57_ (Supplemental Figure 1, A and B). Some monohexyl CM (MHCM) species are decreased in M_NOD_, relative to MΦ_C57_ — in particular, C16:0-MHCM (Supplemental Figure 1, C and D). Several sphingomyelin (SM) species (C18:1, C18:0, C20:0, C22:0, and C24:1) were elevated under basal conditions, with the 16:0 species decreasing in the MΦ_NOD_, relative to MΦ_C57_ (Supplemental Figure 1, E and F). The only significant difference under classical activation was an increase in the C24:1-SM in MΦ_NOD_, relative to MΦ_C57_. Among the CM-1–phosophate (C1P) species, C22:0 was lower and C24:0 higher under basal conditions and 16:0 higher under classical activation in MΦ_NOD_, relative to MΦ_C57_ (Supplemental Figure 1, G and H). Although little is known as to the chain length specificity of C1P in driving inflammatory responses, the C16:0 species is usually associated with inflammatory responses, induction of inflammatory eicosanoid biosynthesis, and MΦ migration (25).

Collectively, these findings suggest that the spontaneous diabetes–prone NOD is inherently in a heightened inflammatory state, as reflected by the higher abundances of proinflammatory lipids and higher iPLA_2_β mRNA (C57, 1.00 ± 0.07; NOD, 1.83 ± 0.05, *P* < 0.001, *n* = 3/group).

### Reduction in iPLA_2_β expression in NOD mitigates T1D parameters and favors M2-MΦ phenotype

**Figure 3:**
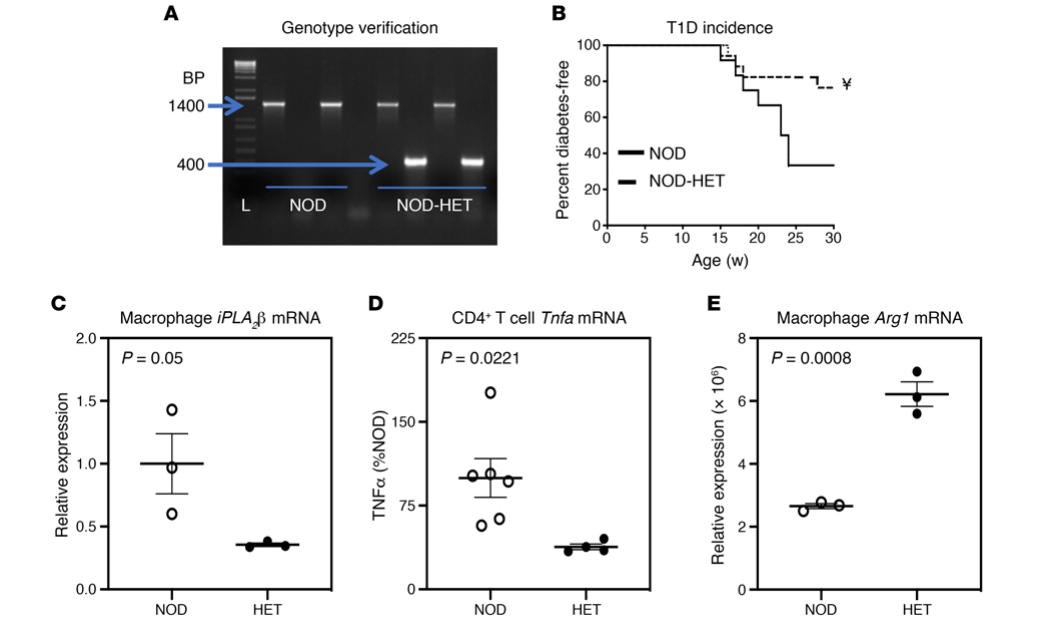
NOD.*iPLA_2_β*^+/–^ genotype and diabetes phenotype. (**A**) Genotype. DNA was generated from tail clips and progeny were genotyped by PCR analyses. Reactions were performed in the presence of primers for the WT sequence (NOD) or for the disrupted sequence (NOD-HET) for each mouse. The expected bands for the WT (1400 bp) and HET (1400 and 400 bp) in 2 mice each are presented. L, bp ladder. (**B**) T1D incidence. Blood glucose was monitored weekly for up to 30 weeks, and 2 consecutive readings of ≥ 275 mg/dL were recorded as onset of diabetes (*n* = 12 and 17 for NOD and NOD-HET groups, respectively). NOD-HET significantly different from NOD; **^¥^***P* < 0.001. (**C**) RNA was isolated from NOD (*n* = 3) and NOD-HET (*n* = 3) macrophages and cDNA prepared for *iPLA_2_β* mRNA analyses by qPCR. (**D**) Production of TNF-α by CD4^+^ T cells. Splenocytes were prepared from the NOD and NOD-HET, and CD4^+^ T cells were isolated and activated, as described in Methods. The media was collected at 72 hours, and TNF-α concentration was determined by ELISA (*n* = 3 per group). (**E**) RNA was isolated from NOD (*n* = 3) and NOD-HET (*n* = 3) macrophages and cDNA prepared for *Arg1*. Statistical analyses: (**B**) Mantel-Cox test; (**C–E**) Student’s *t* test.

As the elevated lipids in MΦ_NOD_ can be generated in an iPLA_2_β-dependent manner, we examined the consequences of reduced iPLA_2_β expression on T1D development by comparing NOD and NOD-HET littermates. Genotype was verified by PCR analyses (Figure 3A), which generated the expected product sizes of 1400 bp for NOD and 1400 bp and 400 bp for NOD-HET. Blood glucose monitoring revealed approximately 75% T1D incidence in NOD (Figure 3B). In contrast, approximately 80% of the NOD-HET remained diabetes free, and this was accompanied by reduced *iPLA_2_β* (~65%) (Figure 3C) and TNF-α production by CD4^+^ T cells (Figure 3D) and higher M2 marker, *Arg1* (Figure 3E), relative to NOD. Furthermore, insulitis was reduced in the NOD-HET (20%–24%) relative to NOD (49%–56%). These findings support a link between iPLA_2_β, MΦ_NOD_ polarization, and T1D development, raising the importance of identifying the iDLs contributing to T1D development.

### Reduced iPLA_2_β expression mitigates MΦ_NOD_ production of select proinflammatory lipids

#### Eicosanoids and SPMs.

**Figure 4:**
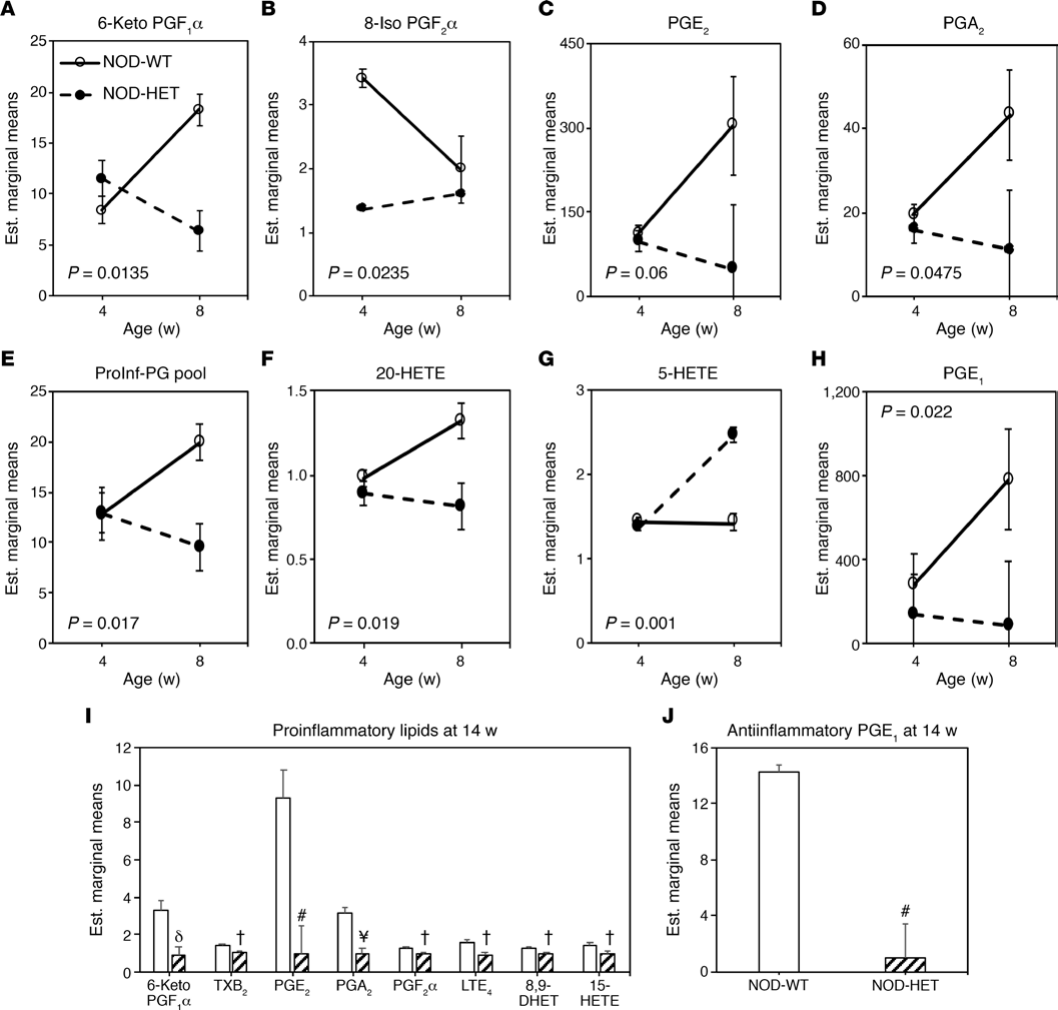
Comparison of eicosanoid production by MΦ_NOD_ and MΦ_NOD-HET_. Peritoneal MΦ isolated from female NOD and NOD-HET mice were treated with vehicle control (DMSO) or classically activated with IFN-γ + LPS, and the media was collected for eicosanoid analyses at 16 hours. The data (estimated marginal mean ± SEM) represent fold-change in activated lipids, relative to corresponding control. MΦ_NOD_ (*n* = 9 and 5) and MΦ_NOD-HET_ (*n* = 4 and 3) at 4 and 8 weeks, respectively. (**A**) 6-Keto PGF_1_α. (**B**) 8-Iso PGF_2_α. (**C**) PGE_2_. (**D**) PGA_2_. (**E**) Proinflammatory prostaglandin (PG) pool. (**F**) 20-HETE. (**G**) 5-HETE. (**H**) PGE_1_. (**I** and **J**) Proinflammatory (**I**) and antiinflammatory PGE_1_ (**J**) at 14 weeks. NOD-HET significantly different from NOD, **^†^***P* < 0.05; ^δ^*P* < 0.01; **^#^***P* < 0.005; **^¥^***P* < 0.001, *n* = 9 in each group. Statistical analyses: (**A–H**) multivariate 2-way ANOVA and time-course ANOVA; (**I** and **J**) Student’s *t* test.

In view of the above observations, we predicted that decreased iPLA_2_β expression would mitigate production of proinflammatory lipids by MΦ between 4 and 8 weeks of age. Production of lipids by MΦ_NOD_ and MΦ_NOD-HET_ under classical activation was not significantly different at 4 weeks of age (Figure 4), with the exception of 8-Iso PGF_2_α, which was higher from MΦ_NOD_, relative to MΦ_NOD-HET_. Between 4 and 8 weeks of age, classical activation resulted in lower production of several proinflammatory lipids (6-keto PGF_1_α, 8-Iso PGF_2_α, PGE_2_, PGA_2_, total proinflammatory pool, and 20-HETE) by MΦ_NOD-HET_ (Figure 4, A–F), relative to MΦ_NOD_. However, production of proinflammatory 5-HETE by MΦ_NOD-HET_ was higher (Figure 4G) and antiinflammatory was PGE_1_ lower (Figure 4H), relative to production by MΦ_NOD_. Interestingly by 14 weeks of age, the production of eicosanoids by MΦ_NOD_ and MΦ_NOD-HET_ was dramatically reduced, but production of several of the same proinflammatory PGs, LTE_4_, (±) 8,9-DHET, and 15-HETE by MΦ_NOD_ remained significantly higher, relative to MΦ_NOD-HET_ (Figure 4I). Moreover, production of PGE_1_ by MΦ_NOD_ continued to be higher, relative to MΦ_NOD-HET_ (Figure 4J; absolute fold increases from independent measures of select lipids are presented in Supplemental Figure 2). All other eicosanoids, SPMs, and fatty acids were not significantly affected between 4 and 14 weeks (Supplemental Table 4, A and B). These findings reveal that production of select proinflammatory eicosanoids is modulated by iPLA_2_β in an age-dependent manner, before the development of hyperglycemia (Supplemental Figure 3).

#### Sphingolipids.

Though classical activation induced changes in the various sphingolipid classes, there were no significant differences in the total pools of CMs, monohexosyl CMs, SMs, CM-1Ps, or sphingosines between MΦ_NOD_ and MΦ_NOD-HET_ (Supplemental Table 4C). These findings suggest that iPLA_2_β-mediated sphingolipid production by MΦ during the prediabetic phase may not be important contributors to T1D development.

### Select plasma lipid changes are associated with iPLA_2_β inhibition or expression

#### NOD versus NOD-HET.

**Figure 5:**
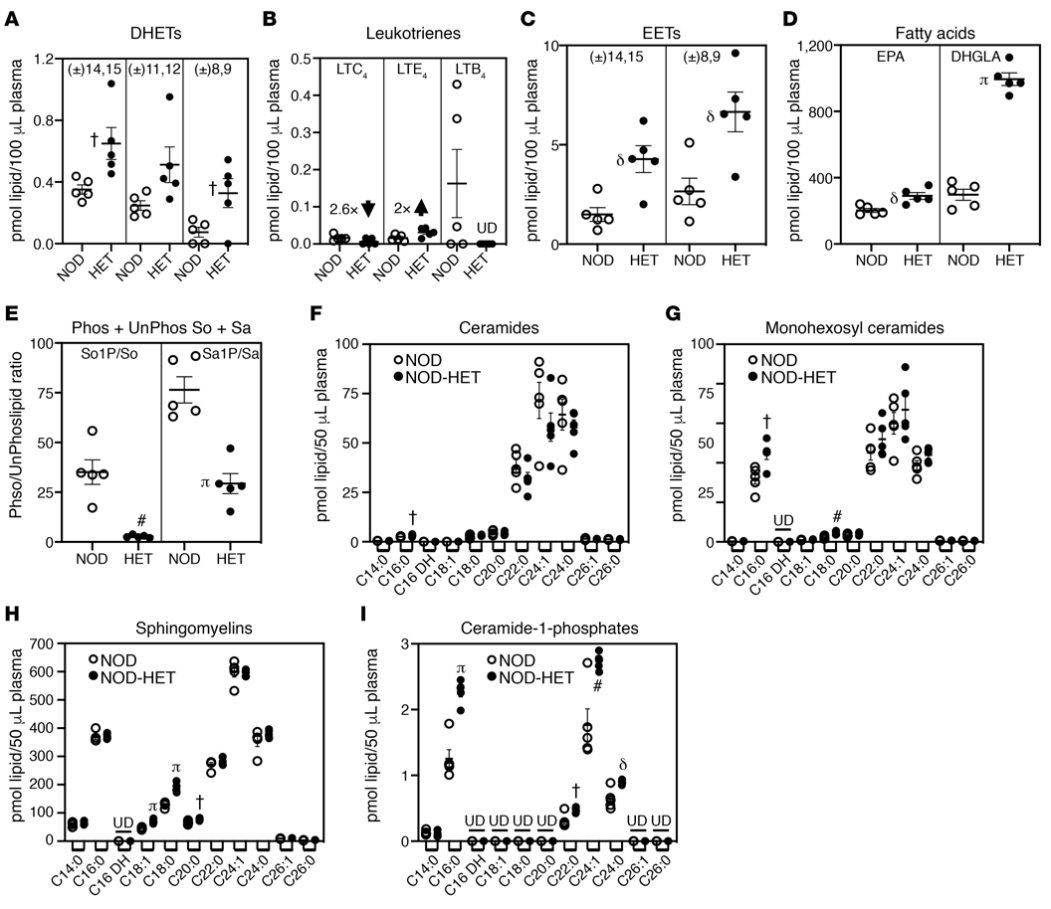
Comparison of select plasma lipids during the prediabetic phase. (**A–I**) Plasma was prepared from NOD (*n* = 5) and NOD-HET (*n* = 5) and processed for lipidomics analyses of eicosanoids (**A–C**), fatty acids (**D**), and sphingolipids (**E–I**). The data (mean ± SEM) represent pmol of each lipid species in 100 μL (**A–D**) or 50 μL (**E–I**) plasma. (**A**) DHETs. (**B**) Leukotrienes. (**C**) EETs. (**D**) EPA and DHGLA. (**E**) Sphingosine and sphinganine (phosphorylated/nonphosphorylated). (**F**) Ceramides. (**G**) Monohexosyl Ceramides. (**H**) Sphingomyelins. (**I**) Ceramide-1-phosphates. NOD-HET significantly different from NOD, **^†^***P* < 0.05; ^δ^*P* < 0.01; **^#^***P* < 0.005; **^π^***P* < 0.0005. Statistical analyses: Student’s *t* test. UD, undetected.

To determine if inhibition of iDL production can also be evidenced in circulating levels of lipids, we assessed plasma lipidome of NOD and NOD-HET through 14 weeks of age (prediabetic phase). At 4 and 8 weeks of age, no significant differences in eicosanoids, sphingolipids, or fatty acids were noted between the NOD and NOD-HET (data not shown). At 14 weeks of age, proinflammatory DHET abundance was low but higher in the NOD-HET, relative to NOD (Figure 5A). Among the proinflammatory LTs, LTC_4_ was reduced 2.6-fold, its precursor LTE_4_ increased 2-fold, and LTB_4_ was absent in NOD-HET, relative to NOD (Figure 5B). In contrast, the abundance of antiinflammatory epoxyeicosatrienoic acids (EETs) were greater and significantly higher in the NOD-HET, relative to NOD (Figure 5C). Moreover, the abundance of EPA and di-homo-γ-linolenic acid (DHGLA) was higher in NOD-HET by 14 weeks of age, as compared with NOD (Figure 5D). Furthermore, the ratios of phosphorylated to nonphosphorylated sphingosine (So1P/So) and sphinganine (Sa1P/Sa) were lower in the NOD-HET by 14 weeks of age, relative to NOD (Figure 5E). Analyses of plasma CM sphingolipids revealed a select increase in CM C16:0 in NOD-HET, relative to NOD at 14 weeks of age (Figure 5F). However, multiple monohexosyl CMs (Figure 5G), SMs (Figure 5H), and CM-1Ps (Figure 5I), including the C16:0 species, were increased in the NOD-HET, relative to NOD.

**Figure 6:**
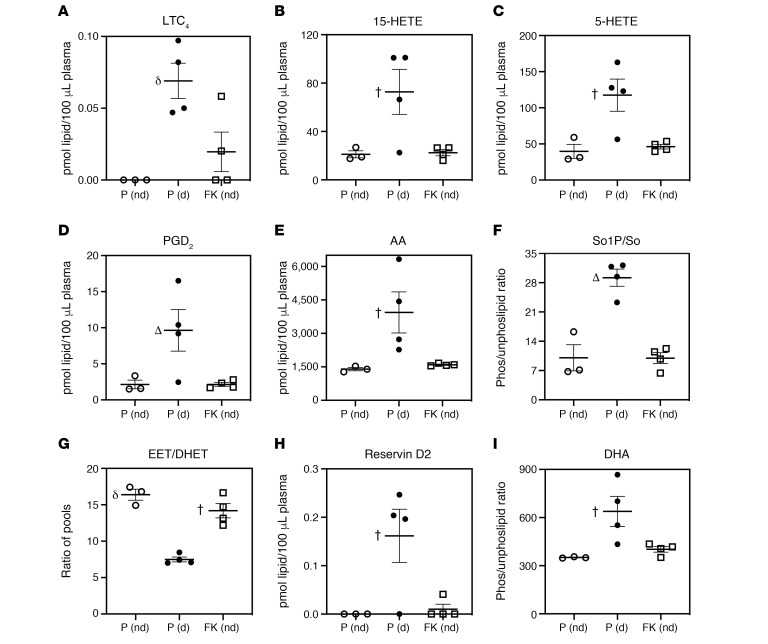
Comparison of select plasma lipids at T1D onset. NOD mice were treated with PBS-T or with FKGK18, starting at 10 days of age, and sacrificed at the onset of T1D (d) or at 30 weeks if they remained nondiabetic (nd). Plasma was prepared from these mice and processed for lipidomics analyses. The data (mean ± SEM) represent pmol of each lipid species in 100 or 50 μL plasma. (**A**) LTC_4_. (**B**) 15-HETE. (**C**) 5-HETE. (**D**) PGD_2_. (**E**) AA. (**F**) So1P/So. (**G**) EET/DHET. (**H**) Resolvin D2. (**I**) DHA. *n* = 3, 4, and 4 for PBS-T (P [nd]), PBS-T (P [d]), and FKGK18 (FK [nd]), respectively. P (d) significantly different from the other groups, **^†^***P* < 0.05; ^δ^*P* < 0.01; **^Δ^***P* < 0.000001. One-way ANOVA.

Next, to determine if a proinflammatory landscape persists until T1D onset, we performed lipidomic analyses with plasma from FKGK18- and PBS-T–treated NOD (starting at 10 days). The analyses comparing PBS-T–treated NOD that did not become diabetic (P [nd]), vehicle-treated NOD that became diabetic (P [d]), and FKGK18-treated (from 10 days of age) NOD that did not turn diabetic (FK [nd]) revealed significantly greater abundance of proinflammatory LTC_4_, 15-HETE, 5-HETE, PGD_2_, and AA in the plasma from diabetic PBS-T–NOD, in comparison with nondiabetic PBS-T– or FKGK18–treated NOD (Figure 6, A–E, respectively). Furthermore, the ratio of So1P/So was higher in diabetic PBS-T–NOD, in comparison with nondiabetic PBS-T– or FKGK18-treated NOD (Figure 6F). In contrast, the ratio of antiinflammatory EET to proinflammatory DHET pools was reduced in diabetic PBS-T–NOD, in comparison with nondiabetic PBS-T– or FKGK18-treated NOD (Figure 6G). Surprisingly, SPM resolvin D2 (Figure 6H) and its fatty acid source DHA (Figure 6I) were significantly higher in the diabetic group, in comparison with either nondiabetic group. Comparison of PBS-T– and FKGK18–treated mice that developed T1D revealed no differences between the two groups (initiated at 10 days or 4 weeks of age), with the exception of a decrease in PGD_2_ in the FKGK18 group, relative to the PBS-T (d) groups (Supplemental Figure 4). Collectively, these analyses reveal select changes in iPLA_2_β-dependent plasma lipid profiles that may be important indicators of T1D development.

### Adoptive transfer of peritoneal iPLA_2_β-deficient MΦ reduces T1D incidence

Adoptive transfer of M2-MΦ_NOD_ has been reported to reduce T1D incidence in the NOD (15). Using an analogous approach, we performed an adoptive transfer study using peritoneal MΦ isolated from NOD and NOD.*iPLA_2_β^–/–^* (KO) mice, rationalizing that the KO MΦ are analogous to M2-MΦ. We found that T1D incidence in NOD administered the KO MΦ was significantly reduced, relative to the mice administered NOD MΦ (Supplemental Figure 5). These studies support the ability of peritoneal MΦ to infiltrate the islets and participate in the pathogenic process of T1D and support the idea that this can be mitigated when MΦ-iPLA_2_β is reduced.

### Plasma lipidome of subjects at high risk for developing T1D

**Figure 7:**
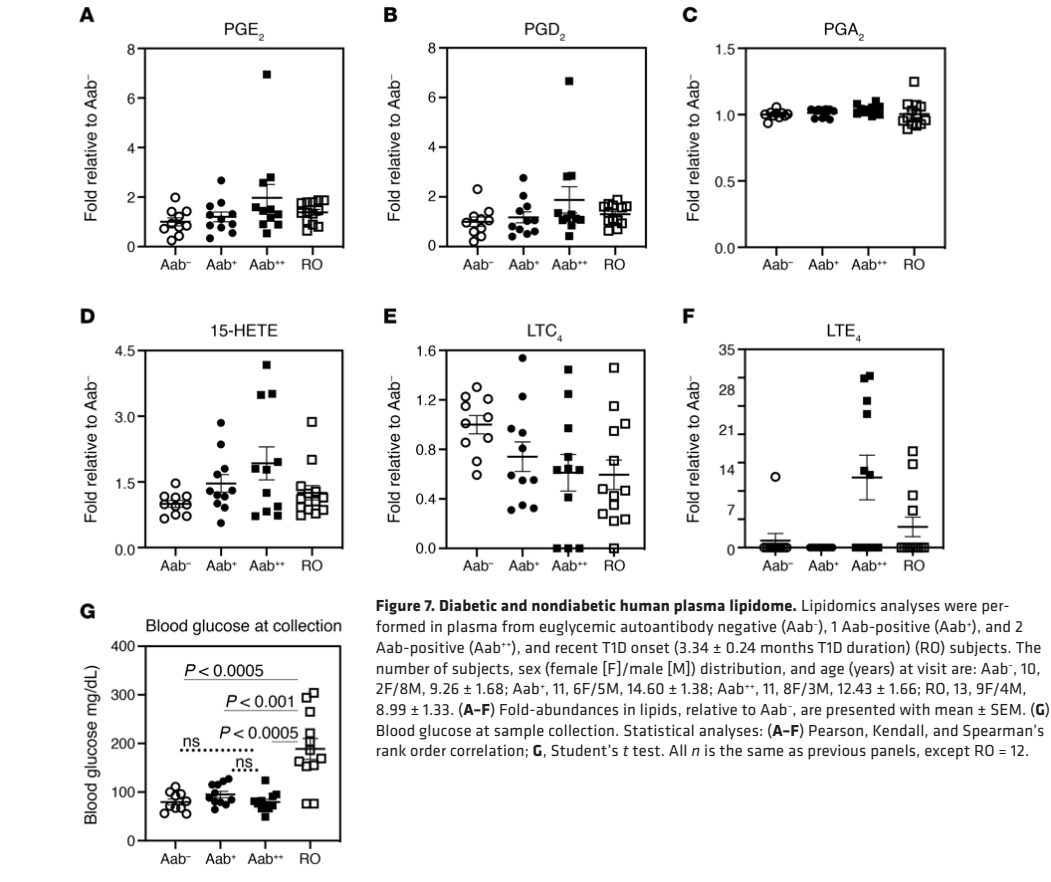
Diabetic and nondiabetic human plasma lipidome. Lipidomics analyses were performed in plasma from euglycemic autoantibody negative (Aab^–^), 1 Aab-positive (Aab^+^), and 2 Aab-positive (Aab^++^), and recent T1D onset (3.34 ± 0.24 months T1D duration) (RO) subjects. The number of subjects, sex (female [F]/male [M]) distribution, and age (years) at visit are: Aab^–^, 10, 2F/8M, 9.26 ± 1.68; Aab^+^, 11, 6F/5M, 14.60 ± 1.38; Aab^++^, 11, 8F/3M, 12.43 ± 1.66; RO, 13, 9F/4M, 8.99 ± 1.33. (**A–F**) Fold-abundances in lipids, relative to Aab^–^, are presented with mean ± SEM. (**G**) Blood glucose at sample collection. Statistical analyses: (**A–F**) Pearson, Kendall, and Spearman’s rank order correlation; **G**, Student’s *t* test. All *n* is the same as previous panels, except RO = 12.

To determine if a similar lipid signature is evident in human subjects at high risk for developing T1D, plasma samples from nondiabetic (normoglycemic) autoantibody negative (Aab^–^), one Aab-positive (Aab^+^), or 2 Aab-positive (Aab^++^) and recent-onset (RO, TID duration < 3.4 months) subjects were processed for lipidomics analyses (Figure 7). The subjects were a mixture of male and female children, between 9 and 15 years old, where no significant differences in prevalence between the sexes are reported (26–28). These assessments identified increases in PGE_2_, PGD_2_, PGA_2_, 15-HETE, and LTE_4_, and a decrease in precursor LTC_4_, in the Aab^++^ group that were significant (*P* < 0.05) or approached significance (Figure 7, A–F), as reflected by Pearson, Kendall, and Spearman rank order correlation analyses (Table 1). Notably, differences recorded in the Aab^++^ subjects occurred in the absence of hyperglycemia (Figure 7G). Of import, these select proinflammatory eicosanoids exhibited a similar step-wise profile: Aab^–^ < Aab^+^ < Aab^++^, with the RO group trending back to Aab^–^ levels. Taken together with the observations in the NOD models, these findings are consistent with a heightened select proinflammatory iDLs landscape in human subjects at high risk for developing T1D.

## Discussion

MΦ are among the first to infiltrate islets and initiate the sequelae of events that cause β cell destruction, and evidence points to involvement of signals generated by immune cells and islets in amplifying the immune responses (29). Very little is known about the contribution of lipid signaling toward β cell death in T1D. We find that, in comparison of spontaneous T1D-prone (NOD) and spontaneous T1D-resistant (C57) mice, MΦ_NOD_ produce significantly higher proinflammatory eicosanoids and sphingolipids under both basal and activated conditions. To date, these are the first demonstrations to our knowledge of a heightened proinflammatory lipid profile in NOD, which would be expected to confer an inherent susceptibility of the NOD to inflammation-mediated responses associated with T1D development.

iPLA_2_β has been reported to participate in a variety of biological processes and contribute to the onset and/or progression of inflammatory disorders (30). Therefore, development of inhibitors of iPLA_2_β has been pursued to counter its deleterious effects. Earlier inhibitors of iPLA_2_β included arachidonyl trifluoromethyl ketone (ATFMK) and bromoenol lactone (BEL); while ATFMK has been shown to also inhibit cytosolic PLA_2_ (cPLA_2_) (31, 32), BEL has been reported to inhibit a variety of enzymes with active site cysteine residues (33). This prompted efforts to develop more selective inhibitors of iPLA_2_β and led to the initial identification of FKGK11 (1,1,1,2,2-pentafluoro-7-phenylheptan-3-one) (34). Continued structure-activity relationship analyses revealed that a trifluoromethyl ketone compound, FKGK18, exhibited much greater selectivity and potency toward iPLA_2_ than cPLA_2_ or secretory PLA_2_ (sPLA_2_) (35). Subsequently, we demonstrated that FKGK18 was a reversible inhibitor with greater selectivity for iPLA_2_β than iPLA_2_γ. Furthermore, FKGK18 was found to be effective in inhibiting ER stress–induced β cell apoptosis, without apparent nonspecific protease activity (36). Our body of previous work suggested a role for iPLA_2_β in β cell apoptosis, which leads to T1D development; we therefore considered the possibility that iPLA_2_β inhibition with FKGK18 might lead to mitigation of T1D incidence. Guided by an earlier report demonstrating beneficial effects of FKGK11 in experimental autoimmune encephalomyelitis (EAE) (10), we investigated the effects of FKGK18 in the spontaneous T1D–prone NOD mouse. We found that FKGK18 administration to NOD starting at 10 days of age ameliorated insulitis and T1D incidence, without promoting nonspecific cytotoxic effects (8). These findings motivated us to use FKGK18 to further investigate the temporal impact of iPLA_2_β on T1D development.

From the studies here, we were able to glean that iPLA_2_β inhibition starting at 4 weeks (age of insulitis onset), but not at 8 weeks (heightened inflammation), reduced NOD diabetes incidence. However, the 8-week regimen promoted some beneficial endotypes, although they were muted in comparison with neonatal inhibition (8). No beneficial outcomes were evident on T1D incidence when FKGK18 was withdrawn at 14 weeks of age (initiated at 10 days). These data stand in contrast to the continued protection against development of symptoms associated with EAE following withdrawal of FKGK11 (10). This raises the possibility that the presence of persistent stressors in spontaneous models of autoimmune disease makes the mice more susceptible to treatment withdrawal, in comparison with an induced EAE model. Consistently, we find that continual iPLA_2_β inhibition (8) or genetic iPLA_2_β reduction was effective in dramatic and equivalent amelioration in T1D. These findings led us to posit that the impact of iDLs on T1D development is in the prediabetic phase and provided motivation to pursue expanded lipidomics analyses in genetically modified NOD, thus precluding potential nonspecific effects of chemical inhibitors.

We subsequently generated NOD with reduced expression of iPLA_2_β (NOD.*iPLA_2_β^+/–^* or NOD-HET) and sought to determine if there was a correlation between iPLA_2_β expression, MΦ_NOD_ lipidome, and the course of T1D development. At 4 weeks of age, activated production of various eicosanoids and sphingolipids by MΦ_NOD_ and MΦ_NOD-HET_ was similar. Sphingolipid production remained similar between the 2 groups through 14 weeks of age, suggesting a lesser impact of iPLA_2_β in MΦ sphingolipid metabolism. In contrast, by 8 weeks of age, production of select proinflammatory PGs (8-iso PGF_2_α, PGE_2_, PGA_2_, proinflammatory PG pool) and HETEs (20-HETE and 5-HETE) by MΦ_NOD_ was significantly higher than by MΦ_NOD-HET_. This is consistent with the higher iPLA_2_β expression in prediabetic MΦ_NOD_ and the predominant antiinflammatory M2 phenotype of M_NOD-HET_. The select, and not universal, changes in the lipidome highlights the potential impact of these iDLs during the prediabetic phase, when ER stress and cytokines, known inducers of iPLA_2_β (37), are ramped up in the NOD (38, 39). At 14 weeks, corresponding to the age approaching T1D onset, lipid production by both MΦ_NOD_ and MΦ_NOD-HET_ was dramatically reduced. However, production of several proinflammatory lipids by MΦ_NOD_ remained higher than by MΦ_NOD-HET_. Surprisingly, production of antiinflammatory PGE_1_ from DHGLA by MΦ_NOD_ continued to be higher than by MΦ_NOD-HET_, likely reflecting attempts at resolution and the dramatic drop from 8 weeks of age, a failure to overcome the burden of inflammation. Taken together, with the loss of protective effects of FKGK18 following its withdrawal, these findings support the presence of persistent stressors in spontaneous models of autoimmune disease, which renders them more susceptible to treatment withdrawal. In the presence of iPLA_2_β-dependent changes in MΦ_NOD_ lipidome during T1D development, we considered the possibility that this may also be reflected in the plasma. Assessment of plasma lipidome during the prediabetic phase revealed no differences at 4 and 8 weeks of age. However, significant differences in select lipids were noted by 14 weeks of age. These included lower DHETs but greater decreases in EETs, higher LTC_4_ (activates NOX4 and induces ROS production) (40) with a corresponding decrease in its precursor LTE_4_ (inflammatory through GPR99) (41), and detection of LTB_4_ (potent chemoattractant and induces ROS) in the NOD-WT, relative to NOD-HET. The EETs are generated via CYP-catalyzed metabolism of arachidonic acid and have antiinflammatory properties (42). However, they can be converted by soluble epoxide hydrolase (sEH1) to DHETs, which are proinflammatory, and the present findings suggest that sEH1 is induced in the NOD, causing a decrease in the EET/DHET ratio. Among the sphingolipids, C16:0 CM was significantly elevated in the plasma of NOD-HET; however, this was associated with increases in the MHCM-C16:0 (and C18:0) and C1P-C16:0. Several species of SMs (C18:1, C18:2, C20:0) and C1Ps (C22:0, C24:1, C24:0) were also higher in the NOD-HET. CMs are considered to be proapoptotic (43); however, their toxicity may be reduced by conversion to monohexosyl CMs, CM-1-phosphates, or SMs. Our findings therefore suggest that reduced expression of iPLA_2_β favors sphingolipid biosynthesis toward species that favor cell survival and that this is reflected in plasma abundances. Furthermore, the ratios of So1P and Sa1P, relative to their nonphosphorylated forms, were markedly lower in the NOD-HET. So1P has been reported to promote T cell migration and retention in inflamed tissues (44). Reduced So1P would be expected to decrease T cell participation in promoting inflammation and is consistent with fewer CD4^+^ T cells and B cells in FKGK18-treated NOD islets (8). While little is known about Sa1P’s role in inflammation, it has been reported to bind to the same receptors as So1P (45) and is thus expected to have similar function. Collectively, our analyses suggest that elevations in select lipids (i.e., LTs, EETs, So1P, Sa1P) may be given consideration as candidate biomarkers of pre-T1D.

When the lipidome signature was assessed at T1D onset using plasma from NOD treated with PBS-T or FKGK18 (10-day regimen), elevations in select lipids were again identified in diabetic mice that were not evident in the nondiabetic mice. These included proinflammatory eicosanoids LTC_4_, 15-HETE, 5-HETE, and PGD_2_, as well as fatty acids AA and DHA. Furthermore, the ratio of EET/DHET was lower in the diabetic group, relative to the nondiabetic groups. Abundance of So1P was also higher in diabetic mice, compared with nondiabetic mice. Of note, 10%–15% of FKGK18-treated mice developed T1D, possibly related to higher susceptibility of these mice to immune responses, and their lipidome was similar to vehicle-treated diabetic NOD, strengthening the link between these iDLs and T1D development. These findings suggest that a proinflammatory iDL signature persists until T1D onset.

Another intriguing finding in our studies is that T1D development is accompanied by select elevations in iPLA_2_β-modulated antiinflammatory lipids, including resolvin D2 and its source, DHA; PGE_1_ and its source, DHGLA (46); and EPA, which is a source of E-series resolvins (47), undetected in our analyses. During inflammation, generation of pro- and antiinflammatory factors (i.e., cytokines and chemokines) can occur (48). This appears to be true with lipids, as well, and may reflect triggering of compensatory mechanisms to affect resolution, which — if not reinforced — leads to disease progression and frank diabetes. In T1D, it is likely that the impact of proinflammatory lipids, if not preempted, is far greater than that of resolving lipids, to the extent that resolution of inflammation fails and diabetes ensues (49). Indeed, Serhan’s group reported that resolvins were increased in sepsis patients with lower survival, which is contradictory to function (50). Thus, our findings suggest that resolving lipids may have therapeutic value if given early by providing a break in the onset/progression of inflammation.

Our identification of changes in the abundances of iDLs (i.e., DHETs, LTs, HETEs, So1P, PGE_1_) before T1D onset highlights the possibility that manipulating select lipid signaling during the prediabetes phase could be beneficial in preventing T1D development. Consistently, reduced T1D incidence has been reported in NOD.*Alox15^–/–^* mice (38). While that study did not examine the lipidome, our findings suggest that production of HETEs and LTs during T1D development is modulated by iPLA_2_β. They also raise the possibility that generating or repurposing FDA-approved drugs that inhibit sEH1 (TPPU), downregulate So1P (FTY720), interrupt LT and HETE signaling (receptor antagonists), or mimic the effects of PGE_1_ (Alprostadil) and SPMs (resolvins) could be beneficial in altering the course of T1D onset and/or progression.

One potential drawback of our studies may be that islet-resident MΦ, rather than peritoneal, are the key pool in the pathogenesis of T1D. However, technical challenges prohibit thorough lipidomic analyses of islet-resident MΦ as designed in our study. Elegant studies by the Unanue group revealed the presence of only 10 resident MΦ/islet in the NOD (51, 52). This low number precludes the ability to perform current lipidomics analyses that would require several thousand cells. Circulating monocyte-derived MΦ do comprise a significant source of MΦ infiltrate within the islet (53, 54), and monocytes would be a feasible pool to harvest for examination. However, due to the fact that circulating monocytes require in situ MΦ differentiation to address function, we felt this method would potentially compromise optimal lipid analyses as designed in our studies. Alternatively, peritoneal MΦ are easily retrievable from rodents and can be obtained in sufficiently large numbers from a single mouse to facilitate testing under multiple conditions. As far back as 1994, Shimada et al. reported that administration of peritoneal MΦ from overtly diabetic NOD into young NOD accelerated insulitis and T1D incidence (55). Horio et al. demonstrated that peritoneal exudate that was rich in MΦ promoted ROS production in cultured islets, an effect that was not seen following exposure to T cells (56). Using an adoptive transfer protocol, Parsa et al. (15) isolated bone marrow–derived MΦ and treated them under conditions to generate MΦ of M2 phenotype. NOD were then administered with either untreated or treated MΦ by the i.p. route. Whereas NOD administered untreated MΦ (or PBS alone) exhibited the expected T1D incidence (about 80% by 27 weeks of age), the mice administered M2-MΦ were protected from developing T1D (only about 20%). Fluorescence and immunohistochemical analyses provided evidence of accumulation of the M2-MΦ within the pancreas and in close proximity to islets. Consistently, we find that adoptive transfer of peritoneal MΦ isolated from NOD and NOD.iPLA_2_β^–/–^ (KO) mice reduced T1D incidence in NOD following i.p. administration of peritoneal MΦ from MΦ_NOD-KO_ mice, relative to NOD administered MΦ_NOD_. These findings support the ability of peritoneal MΦ to migrate and infiltrate the pancreas/islets to promote affect and allow us to posit that the findings presented here can be representative of MΦ that impact islets, leading to T1D.

Importantly, we report here a similar lipidome in the plasma of children (<15 years of age) that are at high risk for developing T1D. As per the Scientific Statement of the Juvenile Diabetes Research Foundation (JDRF), Endocrine Society, and American Diabetes Association (ADA) (57), presymptomatic Aab^++^ are at 44% and 70% risk for developing disease within 5 and 10 years, respectively, but with a lifetime risk approaching 100% (58). Examination of the lipidome of euglycemic Aab^–^, Aab^+^, and Aab^++^ children revealed increases in the lipid signature (PGE_2_, PGD_2_, PGA_2_, 15-HETE, and LTE_4_) in the plasma of euglycemic Aab^++^, relative to nondiabetic Aab^–^ and Aab^+^ children. Because these lipids were also among those identified as iDLs in the NOD controls during the prediabetic and T1D onset phases (Table 2), the lipidome identified in plasma from human children at high risk supports the possibility that these iDLs are linked to T1D onset in humans.

Increases in glucose concentrations promote iPLA_2_β-mediated hydrolysis of arachidonic acid from β cell membrane glycerophospholipids (59, 60), and a recent in vitro study suggests that long-term exposure to hyperglycemia sensitizes MΦ to cytokine stimulation (61). These findings raise the possibility that hyperglycemia drives the evolution of altered MΦ lipid profile during T1D development. However, several lines of studies suggest that this is not the case. Niu et al. (62) compared the impact of hyperglycemia in both insulin-requiring diabetes (STZ) and type 2 (*ob/ob*) model on peritoneal MΦ and reported that MΦ from the STZ mice exhibited an increase in the proinflammatory status of the MΦ. In contrast, they found that the peritoneal MΦ from *ob/ob* mice (T2D model) did not exhibit a proinflammatory phenotype, though they were moderately hyperglycemic. They concluded that increased proliferation and infiltration of MΦ, but not hyperglycemia, was responsible for the inflammation associated with diabetes. Kanter et al. (63) assessed MΦ inflammatory phenotype using peritoneal MΦ and monocytes from 2 models of rodent T1D (STZ and low-density lipoprotein receptor expressing a viral glycoprotein), which exhibited hyperglycemia. They found that hyperglycemia increased both the basal and thioglycolate-elicited inflammatory phenotypes in peritoneal MΦ and that this was recapitulated in monocytes, suggesting that the inflammatory profile is similar between peritoneal MΦ and the circulating monocytes, further supporting our study design. Importantly, they also noted a similar inflammatory phenotype in monocytes from human subjects with T1D, suggesting translatability of the proinflammatory landscape. Furthermore, they reported that hyperglycemia promoted increases in peritoneal MΦ production of PGE_2_, PGD_2_, 15-HETE, and PGF_2_α (similar to our findings). While *Ptgs2* and *Ptges* mRNA, which encode COX-2 and PTGS2, were induced by hyperglycemia, other enzymes that metabolize arachidonic acid (5-LO, 15-LO, or thromboxane A synthetase 1) were not in the M1-MΦ. These findings suggest that while hyperglycemia can impact lipid production from MΦ, it is limited to the COX-2 pathway.

To date, the effects of hyperglycemia on MΦ iPLA_2_β have not been examined, to our knowledge. Here, we report that iPLA_2_β mRNA is significantly higher in MΦ from prediabetic (7–8 weeks of age and normoglycemic) NOD female, relative to age-matched C57 spontaneous diabetes–resistant mice. Furthermore, our data reveal increases in NOD MΦ production of lipids before development of hyperglycemia (i.e., between 4 and 8 weeks of age). In fact, as the age of diabetes nears (14 weeks), there are significant decreases in those lipids. Additionally, several lipid species in the plasma of normoglycemic human subjects positive for 2 Aabs (Aab^++^) are higher, relative to Aab^–^ subjects. Taken together, these studies suggest that the lipid production by peritoneal MΦ we are reporting here is not driven by hyperglycemia. Nevertheless, as hyperglycemia can induce lipid production by a variety of cells, including MΦ and β cells, it is likely a mechanism that may maintain high lipid levels after T1D onset.

In summary, we demonstrate that T1D development in the rodent is associated with a heightened inflammatory lipid landscape that evolves during the prediabetic phase. Such findings are also reflected in the plasma of high-risk human subjects, suggesting that monitoring of prediabetic human plasma lipidome will offer guidance for earlier intervention to better counter the T1D development. Importantly, our work identifies critical participation of iPLA_2_β and select iDLs in T1D development, thus identifying potentially novel lipid-signaling candidates that can be targeted for therapeutics and, in conjunction with Aabs, serve as early biomarkers of prediabetes. As such, early interventions to mitigate the inflammatory lipid profile may be beneficial in ameliorating T1D development.

## Methods

Construction of NOD.PLA2G6^-null^/Srvem mice

NOD breeding pairs were obtained from The Jackson Laboratory, and only female progeny, with expected 80% diabetes incidence, were used in experiments. NOD.*iPLA_2_β^+/–^* (NOD-HET) were generated by breeding male NOD with iPLA_2_β-deficient (KO) female C57BL/6J (64), provided by John Turk (WUSM). The investigator-induced–null *PLA2G6* allele was congenically introgressed into the NOD genome by first generating F1 hybrids from outcrosses of C57BL/6J with NOD. These F1 hybrid females were backcrossed to NOD males, and the female progeny of each successive generation were backcrossed to NOD males for a total of 10 generations. To eliminate contaminating chromosomal segments, genotyping was performed by PCR amplification of 94 polymorphic microsatellite primers (Invitrogen) covering all 19 autosomes for the first 6 generations, as described previously (65). By N6, mice were homozygous for NOD genome at all loci, except those in tight linkage with *PLA2G6* on chromosome 15. From N6 until N10, genotyping was performed with markers on chromosome 15 to ensure transmission of the nonfunctional *PLA2G6* allele (65), allowing for mice with the smallest possible congenic segment to be bred. At generation N10, these marker-assisted or speed congenic mice were intercrossed to generate mice that were homozygous for the *PLA2G6^-null^* allele. These mice were then bred to generate NOD-WT (NOD) and NOD.*iPLA_2_β^+/–^* (NOD-HET) littermates used in subsequent studies.

### NOD genotyping

Prior to experimentation, the mice were genotyped, as described (23), using the following primers (5′-3′) with expected product sizes: (sense/antisense: AGCTTCAGGATCTC-ATGCCCATC/CTCCGCTTCTCGTCCCTCATGGA, 1400 bp; MaExAS/Neo; GGGGCCTCAGACTGGGA-ATC/TCGCCTTCTATCGCCTTCTTGAC, 400 bp). Data from each genotype were compared against their corresponding WT littermates. The mice were maintained with a standard light/dark cycle with ad libitum access to food and water.

### Animal treatment, monitoring, and assessments

Blood glucose levels were measured weekly via tail vein blood draw (2 μL) with Breeze 2 Blood Glucose Monitoring System (Bayer HealthCare). Diabetes incidence was based on 2 consecutive blood glucose readings ≥ 275 mg/dL, at which time the mouse was euthanized. Experimental groups included the following: (a) FKGK18, a reversible selective inhibitor of iPLA_2_β (36) was administered via *i.p*. injection 3 times/week to NOD at 20 mg/kg body weight starting at 10 days, 4 weeks, or 8 weeks of age for up to 30 weeks, and mice receiving i.p. PBS + 5% Tween 80 (PBS-T) with the same dosing schedule were included as a vehicle control group; (b) NOD were treated with PBS-T or FKGK18 from 10 days to 14 weeks of age and then monitored for 30 weeks; (c) NOD and NOD-HET littermates were monitored for up to 30 weeks of age; and (d) NOD and NOD-HET mice were sacrificed at 4, 8, or 14 weeks of age for lipidomics analyses. For insulin measurements, blood was collected at sacrifice (nonfasting) into BD Microtainer Tubes with serum separator and processed for ELISA (Mercodia Kit). Other assessments, as described (8), included i.p. glucose tolerance test (IPGTT), islet infiltration, immunofluorescence analyses, β cell area, urine PGEM analyses, and CD4^+^ T cell assays. Pancreas section and islet images were captured on an Olympus IX81 microscope using cellSens Dimension software and analyzed using ImageJ software (NIH).

### Isolation and activation of peritoneal MΦ

Mice were euthanized by CO_2_ inhalation and cervical dislocation. Peritoneal MΦ were obtained by filling the peritoneal cavity with cold 5 mL PBS containing 2% FBS, massaging gently, and withdrawing the cell-containing solution. Classical activation (IFN-γ + LPS) experiments were performed with freshly isolated and expanded peritoneal MΦ, as described (18). Briefly, MΦ were treated with 15 ng/mL recombinant IFN-γ (R&D Systems, 485-MI-100) for 8 hours in growth medium followed by addition of 10 ng/mL ultrapure LPS (InvivoGen, tlrl-3pelps) and incubated for 16 hours at 37°C or IL-4 (R&D Systems, 404-ML-010) for 16 hours in growth medium. Naive MΦ, which received no activation stimuli, were maintained in growth medium with no additional treatment. Subsequently, the media and MΦ were collected for analyses of eicosanoid and sphingolipid classes of lipids, respectively.

### MΦ mRNA target analyses

MΦ cultured in 60 mm nontissue culture–treated dishes were lysed in 1 mL of TRIzol (Invitrogen, 15596-026). Total RNA was prepared and purified using RNeasy Mini Kits (QIAGEN, 74104), and 1 μg RNA was converted to cDNA using the Superscript III first-strand synthesis system (Invitrogen, 18080-051), according to manufacturer’s instructions. The cDNA was diluted 10-fold and used as template in conventional or quantitative PCR (qPCR). cDNA transcripts were amplified, as described (23), with the following forward/reverse primers (5′-3′) at T_m_: *PLA2G6*_qRT, GGCAGAAGTGGACACCCCAA/CATGGAGCTCAGGATGAACGC, 60°C; *msARG1*_qRT, AGCACTGAG-GAAAGCTGGTC/CAGACCGTGGGTTCTTCACA, 60°C; and *18S*-qRT, CGCTTCCTTACCTGGTTGAT/ TCCCTCTCCGGAATCGAA, 60°C. qPCR was carried out using SYBR Select Mastermix (Invitrogen, 4472908) according to manufacturer’s instructions using *18S* as an internal control. Relative gene expression levels were determined using the 2^–ΔΔCt^ method.

### Lipidomics analyses

#### Eicosanoids preparation.

Eicosanoids were extracted using a modified extraction process, as previously described (66, 67). Plasma (150 μL) was combined with 850 μL of liquid chromatography–mass spectrometry H_2_O, followed by the addition of an internal standard (IS) mixture. For media analysis, IS was added to media from cells (2 mL). Eicosanoid IS was comprised of 10% methanol (100 μL and 200 μL, respectively, for plasma and media), glacial acetic acid (5 μL and 10 μL, respectively, for plasma and media), and internal standard (20 μL) containing the following deuterated eicosanoids (1.5 pmol/μL, 30 pmol total; all standards purchased from Cayman Chemicals): (d_4_) 6-keto PG F_1_α, (d_4_) PG F_2_α, (d_4_) PGE_2_, (d_4_) PG D_2_, (d_8_) 5-HETE, (d_8_) 12-HETE, (d_8_) 15-HETE, (d_6_) 20-HETE, (d_11_) 8,9 epoxyeicosatrienoic acid, (d_8_) 14,15 epoxyeicosatrienoic acid, (d_8_) arachidonic acid, (d_5_) eicosapentaenoic acid, (d_5_) docosahexaenoic acid, (d_4_) PG A_2_, (d_4_) LT B_4_, (d_4_) LT C_4_, (d_4_) LTD_4_, (d_4_) LTE_4_, (d_5_) 5(*S*),6(*R*)-lipoxin A4, (d_11_) 5-iPF_2_α -VI, (d_4_) 8-iso PG F_2_α, (d_11_) (±) 14,15-DHET, (d_11_) (±) 8,9-DHET, (d_11_) (±) 11,12-DHET, (d_4_) PG E_1_, (d_4_) thromboxane B2, (d_6_) dihomo-γ-linoleic acid, (d_5_) resolvin D2, (d_5_) resolvin D1, (d_5_) maresin 2, and (d_5_) resolvin D3. Samples and vial rinses (5% MeOH; 2 mL) were applied to Strata-X SPE columns (Phenomenex), previously washed with methanol (2 mL) and then dH_2_O (2 mL). Eicosanoids eluted with isopropanol (2 mL) were dried in vacuuo and reconstituted in EtOH/dH_2_O (50:50; 100 μL) before analysis.

#### Sphingolipids preparation.

Cell pellets and plasma (50 μL) were extracted using a modified Bligh Dyer extraction, as previously described (67–69). Samples were spiked with 250 pmol of C1P, SM, CM, and monohexosyl CM (d18:1/12:0 species), and sphingansine, sphinganine, sphingasine-1–phosphate, sphinganine-1–phosphate (d17:0 sphinganine/d17:1 sphingosine) as internal standard (Avanti Polar Lipids). Among the sphingolipids analyzed were CMs (C14:0, C16:0, C18:1, C18:0, C20:0, C22:0, C24:1, C24:0, C26:1, C26:0), monohexyl CMs (C14:0, C16:0, C18:1, C18:0, C20:0, C22:0, C24:1, C24:0, C26:1, C26:0), SMs (C14:0, C16:0, C18:1, C18:0, C20:0, C22:0, C24:1, C24:0, C26:1, C26:0), CM-1-phosphates (C14:0, C16:0, C18:1, C18:0, C20:0, C22:0, C24:1, C24:0, C26:1, C26:0), 18:1-sphingosine (C18:1-So) C18:1–sphingosine-1–phosphate (C18:1-So1P), and C18:1-sphinganine (C18:1-Sa) and C18:1–sphinganine-1–phosphate (C18:1-Sa1P).

#### Analysis of sphingolipids, eicosanoids, and fatty acids by ultra performance liquid chromatography–electrospray ionization–tandem mass spectrometry.

Lipids in the samples were separated using 2 Shimadzu Nexera X2 LC-30AD pumps coupled to a SIL-30AC auto injector, coupled to a DGU-20A5R degassing unit. Sphingolipids, eicosanoids, and fatty acids were analyzed via mass spectrometry using an AB Sciex Triple Quad 5500 Mass Spectrometer. MRM transitions with corresponding declustering potentials, collision energies, entrance potentials, and collision cell exit potentials are shown in Supplemental Tables 1 and 2.

*MΦadoptive transfer*. Peritoneal MΦ were obtained from 8-week-old female NOD and NOD.iPLA_2_β^–/–^ (NOD-KO) mice. The MΦ (2.75 × 10^6^) were administered i.p. to 8-week-old female NOD, and diabetes incidence was recorded through weekly blood glucose monitoring.

#### Human lipidome.

Study subjects were recruited through Children’s Hospital of Wisconsin, and diagnosis of T1D was defined per World Health Organization criteria (70). All RO T1D subjects were positive for > 1 Aab and were drawn from subjects with histories of good glycemic control (HbA1c, 7.53% ± 0.28%). Subjects within the 3 nondiabetic (normoglycemic) sibling groups (Aab^–^, Aab^+^, Aab^++^) were free of known infection at blood collection. Measurements of Aabs targeting GAD, IA2, insulin, and zinc transporter 8 (ZnT8) were as described (71).

### Statistics

Significant difference in T1D incidence was determined by the Mantel-Cox test. For all other analyses, *P* values were determined using either 2-tailed Student’s *t* test (for analysis of 2 groups), multivariate analysis of variance (for analyses testing more than 1 outcome), time-course ANOVA (for temporal lipid analysis), or 1- or 2-way ANOVA (for tests including more than 1 sample group). To assess the relationship between selected eicosanoids in human plasma, the data were subjected to a linear regression analysis (Pearson, Kendall, and Spearman rank-order correlation) using only the Aab^–^, Aab^+^, and Aab^++^ values, under the rationale that RO subjects are already diabetic and usually controlled with therapeutics. Statistical programs used were either SPSS or R; *P* < 0.05 was taken to indicate significant differences.

### Study approval

All animal experiments were conducted according to approved IACUC guidelines at UAB. Human study participants were recruited through the Children’s Hospital of Wisconsin, and samples were collected as described (72). IRB approval (CHW IRB 01–15) was granted for all analyses, and informed consent/assent was obtained from subjects or their parents/legal guardians. Acquisition and analyses of samples was approved by UAB (IRB-100813004).

## Author contributions

AJN designed and performed the MΦ activation and adoptive transfer experiments; DJS performed lipidomics; RNB performed FKGK18 temporal assessments; CLC prepared samples for mass spectrometry; MAP performed statistical analyses; YGT generated, genotyped, and maintained the mice; XL supervised molecular biology analyses; GK synthesized FKGK18; CLG performed genotyping and annotation of genotypes for generating the NOD models, and prepared the graphical abstract; CEM monitored NOD genotype during backcrossing; JK coordinated recruitment and collection of the human samples; MJH provided the human samples and subject data at time of sample collection; CEC was responsible for all aspects of lipidomics analyses; and SR was responsible for the overall design, performance, data collection and analyses, and writing of the manuscript. All coauthors contributed text to their respective sections and provided edits in the manuscript.

## Supplementary Material

Supplemental data

## Figures and Tables

**Table 1:**
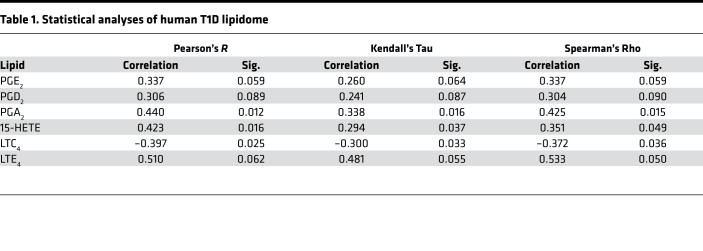
Statistical analyses of human T1D lipidome

**Table 2:**
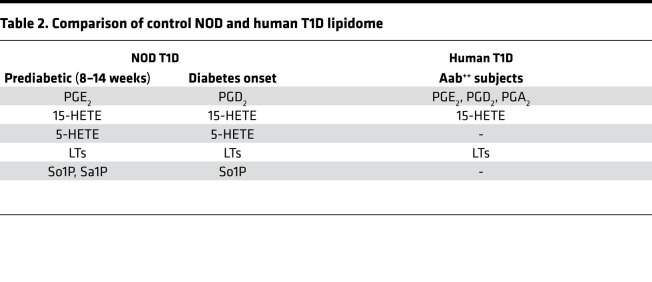
Comparison of control NOD and human T1D lipidome

## Version 1 08/20/2020

Electronic publication
