# Supplementary material for: Lipid mediators and biomarkers associated with type 1 diabetes development
Source: JCI Insight. 2020 Aug 20;5(16):e138034. doi: 10.1172/jci.insight.138034 (PMC7455134; doi:10.1172/jci.insight.138034)
Supplement: Supplemental data [file jciinsight-5-138034-s023.pdf]

SUPPLEMENTAL TABLES

Supplemental Table 1. SCIEX 5500 QTRAP mass spectrometer settings for reverse phase chromatographic separation of various eicosanoids and fatty acids. DP: declustering potential, CE: collision energy, EP: entrance potential, CXP: cell exit potential. Transitions and settings were determined manually *via* direct infusion. Settings and transitions were chosen based on the best signal obtained during infusion using manual tuning in SCIEX Analyst software.

Supplemental Table 2. SCIEX 5500 QTRAP mass spectrometer settings for reverse phase chromatographic separation of various sphingolipids. DP: declustering potential, CE: collision energy, EP: entrance potential, CXP: cell exit potential. Transitions and settings were determined manually *via* direct infusion. Settings and transitions were chosen based on the best signal obtained during infusion using manual tuning in SCIEX Analyst software.

Supplemental Table 3. Eicosanoid production by macrophages from $M\Phi_{NOD}$ and $M\Phi_{c57}$. Peritoneal macrophages were isolated from female NOD (n = 5) and age-matched C57BL/J6 from Jackson Laboratories (n = 12) mice and treated with DMSO vehicle alone (control) or LPS+IFNγ (classical-activation), as detailed in Methods. The media were collected and processed for eicosanoid analyses by UPLC-MS/MS. The data presented are means ± SEMs of ng lipid/10^6 $M\Phi_{NOD}$ under control and activated conditions and the fold abundances in $M\Phi_{NOD}$, relative to $M\Phi_{C57}$. (NOD significantly different from C57: $^{\dagger}p < 0.05$; $^{\partial}p < 0.01$; $^{\#}p < 0.005$; $^{¥}p<0.001$; $^{*}p < 0.0001$; $^{\Delta}p < 0.000001$.)

Supplemental Table 4. Temporal comparisons of eicosanoids, fatty acids, and sphingolipids production by $M\Phi_{NOD}$ and $M\Phi_{NOD\text{-}HET}$. Peritoneal macrophages were isolated from the mice at 4, 8, and 14 weeks of age and treated with either DMSO vehicle alone (control) or IFNγ+LPS (classical-activation). The media was collected and processed for eicosanoids and fatty acids ($M\Phi_{NOD}$, n = 5, 5, and 9; $M\Phi_{NOD\text{-}HET}$, n = 3, 4, and 9 at 4, 8, and 14 weeks of age, respectively) and the cells for sphingolipids ($M\Phi_{NOD}$, n = 5, 5, and 9; $M\Phi_{NOD\text{-}HET}$, n = 3, 4, and 5 at 4, 8, and 14 weeks of age, respectively), by UPLC-MS/MS. The data are estimated marginal means ± SEMs of fold increase in lipids under activating condition, relative to control abundances. (UD = undetected.)

Supplemental Table 1. SCIEX 5500 QTRAP mass spectrometer settings for reverse phase chromatographic separation of various eicosanoids and fatty acids.

Analyte ID	Q1 Mass (Da)	Q2 Mass (Da)	DP (volt)	EP (volt)	CE (volt)	CXP (volt)
6keto PGF1α-d4	373.2	167	-80	-14	-33	-15
6-keto PGF1α	369.2	163	-80	-14	-33	-15
8-iso PGF2α-d4	357.4	197.1	-150	-13	-33	-14
8-iso PGF2α	353.4	193	-130	-13	-31	-11
TXB2-d4	373.2	173	-80	-13	-23	-15
TXB2	369.2	169	-80	-13	-25	-15
5-iPF2α-VI-d11	364.2	115	-90	-12	-28	-20
5-iPF2α-VI	353.2	114.9	-90	-12	-28	-16
PGE2-d9	360.2	280.3	-80	-13	-24	-12
PGE2	351.2	271.2	-80	-13	-25	-12
PGF2α-d9	362.2	193	-70	-10	-35	-18
PGF2α	353.2	193	-70	-10	-31	-18
PGD2-d9	360.201	280.3	-80	-10	-24	-12
PGD2	351.201	271.2	-80	-10	-23	-12
RvD3-d5	380.22	147	-68	-10	-24	-11
RvD3	375.22	147	-68	-10	-24	-11
RvD2-d5	380.2	141	-80	-12	-21	-10
RvD2	375.2	141	-80	-12	-22	-10
PGE1-d4	357.2	239	-105	-13	-20	-19
PGE1	353.2	235	-105	-13	-19	-19
RvD1-d5	380.201	141	-70	-13	-20	-10
RvD1	375.201	141	-70	-13	-22	-10
Lipoxin A4-d5	356.3	114.9	-90	-14	-21	-20
Lipoxin A4	351.3	114.8	-90	-13	-20	-15
PGA2-d4	337.2	275.3	-80	-14	-19	-12
PGA2	333.2	271.2	-80	-14	-19	-12
LTD4-d5	500.3	177	-105	-10	-24	-16
LTD4	495.3	176.9	-105	-10	-19	-14
LTC4-d5	629.3	272.1	-50	-13	-30	-13
LTC4	624.3	272.1	-60	-13	-30	-13
LTE4-d5	443.2	338.3	-80	-10	-24	-15
LTE4	438.2	333.1	-80	-10	-23	-15
LTB4-d4	339.2	197	-95	-14	-21	-16
LTB4	335.2	195	-95	-14	-22	-16
maresin 2-d5	364.23	221.1	-65	-14	-16	-11
maresin 2	359.23	221.1	-65	-14	-16	-11
(±)14,15-DHET-d11	348.2	207	-110	-14	-24	-14
(±)14,15-DHET	337.2	207	-110	-14	-26	-14
15-deoxy-Δ12,14-PGJ2-d4	319.2	275.3	-100	-9	-22	-10
15-deoxy-Δ12,14-PGJ2	315.2	271.2	-100	-9	-21	-10
(±)11,12-DHET-d11	348.2	167	-85	-12	-25	-14
(±)11,12-DHET	337.2	167	-85	-12	-24	-14
(±)8,9-DHET-d11	348.2	185.2	-93	-9	-23	-13
(±)8,9-DHET	337.2	185.2	-95	-10	-20	-13
20-HETE-d6	325.2	281.3	-85	-13	-21	-12
20-HETE	319.2	275.2	-85	-13	-21	-12
15 HETE-d8	327.2	226	-116	-13	-16	-16
15 HETE	319.2	219	-116	-13	-19	-16
12 HETE-d8	327.2	184.1	-90	-13	-20	-16
12 HETE	319.2	178.9	-90	-13	-19	-16
(±)14(15)-EET-d11	330.2	219.1	-90	-13	-15	-15
(±)14(15)-EET	319.2	219.1	-90	-13	-15	-15
5 HETE d8	327.2	116	-90	-13	-18	-10
5 HETE	319.2	115	-90	-13	-20	-10
(±)8(9)-EET-d11	330.2	123	-90	-12	-18	-11
(±)8(9)-EET	319.2	123	-90	-12	-18	-11
EPA d5	306.2	262.3	-86	-10	-15	-18
EPA	301.2	257.1	-86	-10	-17	-18
DHA-d5	332.2	288.3	-95	-12	-14	-12
DHA	327.2	283.2	-95	-12	-19	-12
AA-d8	311.2	267.3	-150	-13	-18	-16
AA	303.2	259.2	-150	-13	-17	-14
DHGLA-d6	311.2	267.2	-105	-14	-20	-13
DHGLA	305.2	261.2	-90	-10	-43	-16

Analyte ID	Q1 Mass (Da)	Q2 Mass (Da)	DP (volt)	EP (volt)	CE (volt)	CXP (volt)
d17:1 So	286.4	268.3	120	10	15	10
d17:0 Sa	288.4	270.4	120	10	21	10
d18:1 So	300.5	282.3	120	10	21	10
d18:0 Sa	302.5	284.3	120	10	21	10
d17:1 So1P	366.4	250.4	120	10	23	10
d17:0 Sa1P	368.4	252.4	120	10	23	10
d18:1 So1P	380.4	264.4	120	10	25	10
d18:0 Sa1P	382.4	266.4	120	10	25	10
C12 Cer	482.6	264.4	80	10	41	10
C14 Cer	510.7	264.4	80	10	43.5	10
C16 Cer	538.7	264.4	80	10	46	10
C18:1 Cer	564.7	264.4	80	10	48.5	10
C18:0 Cer	566.7	264.4	80	10	48.5	10
C20 Cer	594.7	264.4	80	10	51	10
C22 Cer	622.8	264.4	80	10	53.5	10
C24:1 Cer	648.9	264.4	80	10	56	10
C24 Cer	650.9	264.4	80	10	56	10
C26:1 Cer	676.9	264.4	80	10	58.5	10
C26 Cer	678.9	264.4	80	10	58.5	10
C12 C1P	562.4	264.4	80	10	41	10
C14 C1P	590.4	264.4	80	10	43.5	10
C16 C1P	618.5	264.4	80	10	46	10
C18:1 C1P	644.5	264.4	80	10	48.5	10
C18:0 C1P	646.5	264.4	80	10	48.5	10
C20 C1P	674.4	264.4	80	10	51	10
C22 C1P	702.7	264.4	80	10	53.5	10
C24:1C1P	728.6	264.4	80	10	56	10
C24 C1P	730.6	264.4	80	10	56	10
C26:1 C1P	756.7	264.4	80	10	58.5	10
C26 C1P	758.7	264.4	80	10	58.5	10
C12 MonHex	644.6	264.4	80	10	41	10
C14 MonHex	672.6	264.4	80	10	43.5	10
C16 MonHex	700.7	264.4	80	10	46	10
C18:1 MonHex	726.7	264.4	80	10	48.5	10
C18:0 MonHex	728.7	264.4	80	10	48.5	10
C20 MonHex	756.7	264.4	80	10	51	10
C22 MonHex	784.8	264.4	80	10	53.5	10
C24:1 MonHex	810.9	264.4	80	10	56	10
C24 MonHex	812.9	264.4	80	10	56	10
C26:1 MonHex	838.9	264.4	80	10	58.5	10
C26 MonHex	840.9	264.4	80	10	58.5	10
C12 SM	647.7	184.4	80	10	41	10
C14 SM	675.7	184.4	80	10	43.5	10
C16 SM	703.8	184.4	80	10	46	10
C18:1 SM	729.8	184.4	80	10	48.5	10
C18:0 SM	731.8	184.4	80	10	48.5	10
C20 SM	759.9	184.4	80	10	51	10
C22 SM	787.9	184.4	80	10	53.5	10
C24:1 SM	813.9	184.4	80	10	56	10
C24 SM	815.9	184.4	80	10	56	10
C26:1 SM	841.9	184.4	80	10	58.5	10
C26 SM	843.9	184.4	80	10	58.5	10

Supplemental Table 2. Mass spectrometer settings for reverse phase chromatographic separation of sphingolipids.

Supplemental Table 3A. Proinflammatory Eicosanoids Production by MΦ_{NOD}, Relative to MΦ_{C57}

Lipid	Basal		[b]IFNγ + LPS	
	(pmol/10^6 MΦ_{NOD})	[a]Fold (Rel. to MΦ_{C57})	(pmol/10^6 MΦ_{NOD})	[a]Fold (Rel. to MΦ_{C57})
6-keto $PGF_1\alpha$	11.750 ± 3.436[*]	6.306 ± 1.844	80.713 ± 5.252[Δ]	2.785 ± 0.181[Δ]
TXB_2	6.619 ± 0.297[†]	1.529 ± 0.069	10.143 ± 0.328	0.936 ± 0.030
PGD_2	0.278 ± 0.037	0.696 ± 0.092	0.630 ± 0.060[†]	0.025 ± 0.002
8-Iso $PGF_2\alpha$	1.951 ± 0.160[Δ]	23.958 ± 1.967	6.312 ± 0.284[Δ]	27.695 ± 1.247
5-IPFα-VI	0.440 ± 0.078[†]	1.764 ± 0.312	0.682 ± 0.012[#]	2.111 ± 0.036
PGE_2	1.607 ± 0.132	1.831 ± 0.150	165.684 ± 6.148[#]	2.235 ± 0.083
PGA_2	0.853 ± 0.121[#]	2.257 ± 0.320	15.176 ± 0.618[†]	1.880 ± 0.077
15-deoxy-Δ12,14-PGJ_2	0.515 ± 0.048[†]	2.854 ± 0.268	0.992 ± 0.068[#]	2.341 ± 0.16
LTD_4	0.257 ± 0.044[¥]	5.273 ± 0.908	0.154 ± 0.011[∂]	3.017 ± 0.216
LTC_4	0.053 ± 0.026[†]	2.940 ± 1.423	0.043 ± 0.013	1.731 ± 0.536
LTE_4	0.178 ± 0.067[†]	2.520 ± 0.946	0.069 ± 0.003	0.492 ± 0.023
LTB_4	0.586 ± 0.023	1.602 ± 0.062	0.690 ± 0.048	1.236 ± 0.087
20-HETE	22.803 ± 1.552	0.717 ± 0.049	22.644 ± 0.847	0.781 ± 0.029
15-HETE	4.051 ± 0.245	1.020 ± 0.062	10.709 ± 0.366	1.211 ± 0.041
12-HETE	40.970 ± 0.759	1.398 ± 0.026	48.677 ± 0.362[†]	1.441 ± 0.011
5-HETE	4.655 ± 0.068	0.964 ± 0.014	6.573 ± 0.074	1.139 ± 0.013
(±)14,15-DHET	1.875 ± 0.034	0.989 ± 0.018	2.085 ± 0.150	1.196 ± 0.086
(±)11,12-DHET	0.479 ± 0.039	1.320 ± 0.107	0.606 ± 0.047	1.450 ± 0.114
(±)8,9-DHET	0.843 ± 0.054	1.815 ± 0.117	1.191 ± 0.049[†]	2.124 ± 0.087

Supplemental Table 3B. Anti-inflammatory Eicosanoids Production by MΦ_{NOD}, Relative to MΦ_{C57}

Lipid	Basal		[b]IFNγ + LPS	
	(pmol/10^6 MΦ_{NOD})	[a]Fold (Rel. to MΦ_{C57})	(pmol/10^6 MΦ_{NOD})	[a]Fold (Rel. to MΦ_{C57})
$PGF_2\alpha$	0.846 ± 0.134	0.421 ± 0.067	2.518 ± 0.152	0.321 ± 0.019
PGE_1	0.190 ± 0.082	1.572 ± 0.678	28.501 ± 0.901[†]	1.884 ± 0.060
Resolvin D2	0.0185 ± 0.004	0.070 ± 0.016	0.0174 ± 0.005	0.031 ± 0.01
Resolvin D1	0.160 ± 0.010	1.410 ± 0.089	0.176 ± 0.011	1.533 ± 0.098
Lipoxin A4	0.102 ± 0.045	0.581 ± 0.256	0.143 ± 0.043	0.774 ± 0.232
(±)14,15-EET	0.539 ± 0.007	0.766 ± 0.01	0.863 ± 0.044	1.051 ± 0.053
(±)8,9-EET	0.423 ± 0.050	1.294 ± 0.153	0.978 ± 0.096	1.771 ± 0.175

Supplemental Table 3C. Fatty Acids Production by MΦ_{NOD}, Relative to MΦ_{C57}

Lipid	Basal		[b]IFNγ + LPS	
	(pmol/10^6 MΦ_{NOD})	[a]Fold (Rel. to MΦ_{C57})	(pmol/10^6 MΦ_{NOD})	[a]Fold (Rel. to MΦ_{C57})
EPA	134.545 ± 2.625	0.936 ± 0.018	209.022 ± 2.095	1.365 ± 0.014
DHA	769.849 ± 9.955	0.740 ± 0.010	902.551 ± 13.428	0.886 ± 0.013
AA	5355.159 ± 244.850	0.895 ± 0.041	7715.520 ± 61.79	0.950 ± 0.008

Supplemental Table 4A. Classically-Activated Eicosanoids Production by $M\Phi_{NOD}$ and $M\Phi_{NOD-HET}$

Lipid	$M\Phi_{NOD}$			$M\Phi_{NOD-HET}$		
	**4 weeks (n = 9)**	**8 weeks (n = 5)**	**14 weeks (n = 9)**	**4 weeks (n = 4)**	**8 weeks (n = 3)**	**14 weeks (n = 9)**
TXB_2	1.568 ± 0.094	1.730 ± 0.134	1.415± 0.091	1.438 ± 0.121	1.510 ± 0.173	1.026 ± 0.091
5-iPF$_{2}\alpha$-VI	1.566 ± 0.168	1.429 ± 0.289	1.102 ± 0.057	1.059 ± 0.217	1.165 ± 0.373	1.034 ± 0.057
15-deoxy-Δ12,14-PGJ_2	1.937 ± 0.199	2.546 ± 1.195	UD	1.089 ± 0.257	1.130 ± 1.542	UD
$PGF_{2}\alpha$	3.270 ± 0.369	2.863 ± 0.753	1.300 ± 0.090	1.834 ± 0.477	2.877 ± 0.972	0.965 ± 0.09
Resolvin D1	1.056 ± 0.100	1.426 ± 0.196	0.940 ±	1.003 ± 0.129	1.271 ± 0.253	0.981 ± 0.019
LTC_4	1.414 ± 0.576	1.241 ± 0.212	UD	1.376 ± 0.744	1.091 ± 0.274	UD
LTE_4	0.547 ± 0.169	1.651 ± 0.449	1.579 ± 0.188	0.559 ± 0.218	1.133 ± 0.580	0.899 ± 0.188
LTB_4	1.185 ± 0.070	1.092 ± 0.232	UD	1.266 ± 0.090	1.311 ± 0.300	UD
(±)14,15-DHET	1.096 ± 0.063	0.949 ± 0.094	1.003 ± 0l04	0.949 ± 0.094	1.058 ± 0.122	0.958 ± 0.04
(±)11,12-DHET	1.203 ± 0.100	2.128 ±0.566	1.105 ± 0.04	1.097± 0.129	1.135 ± 0.731	1.023 ± 0.04
(±)8,9-DHET	1.415 ± 0.061	1.696 ± 0.576	1.254 ± 0.089	0.987 ± 0.079	1.202 ± 0.744	0.973 ±0.089
15 HETE	2.768 ± 0.088	4.646 ± 0.765	1.408 ± 0.142	2.177 ± 0.114	3.052 ± 0.987	0.952 ± 0.142
12 HETE	1.205 ± 0.028	1.888 ± 0.378	1.113 ± 0.111	1.259 ± 0.037	1.662 ± 0.487	0.907 ± 0.111

Supplemental Table 4B. Classically-Activated Fatty Acids Production by $M\Phi_{NOD}$ and $M\Phi_{NOD-HET}$

Lipid	$M\Phi_{NOD}$			$M\Phi_{NOD-HET}$		
	**4 weeks (n = 9)**	**8 weeks (n = 5)**	**14 weeks (n = 9)**	**4 weeks (n = 4)**	**8 weeks (n = 3)**	**14 weeks (n = 9)**
EPA	1.568 ± 0.061	1.568 ± 0.061	1.174 ± 0.261	1.180 ± 0.071	1.744 ± 0.108	1.042 ± 0.261
DHA	1.183 ± 0.016	1.120 ± 0.125	0.953 ± 0.064	1.052 ± 0.020	1.136 ± 0.161	0.946 ± 0.064
AA	1.436 ± 0.053	2.219 ± 0.238	0.766 ± 0.265	1.313 ± 0.068	2.214 ± 0.307	0.856 ± 0.265

Supplemental Table 4C. Classically-Activated Sphingolipids Production by $M\Phi_{NOD}$ and $M\Phi_{NOD-HET}$

Lipid	$M\Phi_{NOD}$			$M\Phi_{NOD-HET}$		
	**4 weeks (n = 9)**	**8 weeks (n = 5)**	**14 weeks (n = 9)**	**4 weeks (n = 4)**	**8 weeks (n = 3)**	**15 weeks (n = 9)**
Total CMs	3.083 ± 1.364	1.937 ± 0.188	1.264 ± 0.185	1.156 ± 1.760	1.496 ± 0.242	1.515 ± 0.278
Total MHCMs	1.533 ± 0.279	2.357 ± 0.469	1.485 ± 0.290	0.999 ± 0.322	2.661 ± 0.542	1.590 ± 0.435
Total SMs	0.961 ± 0.109	1.349 ± 0.147	1.330 ± 0.222	1.014 ± 0.126	1.337 ± 0.169	1.533 ± 0.333
Total C-1-Ps	0.936 ± 0.083	1.021 ± 0.501	0.731 ± 0.124	0.635 ± 0.096	1.922 ± 0.578	0.749 ± 0.186
So1P/So1	47.218 ± 6.420	56.885 ± 9.960	UD	47.753 ± 8.288	51.256 ± 12.859	UD

Supplemental Figure 1. Sphingolipids Production by MΦ_{NOD} and MΦ_{C57}

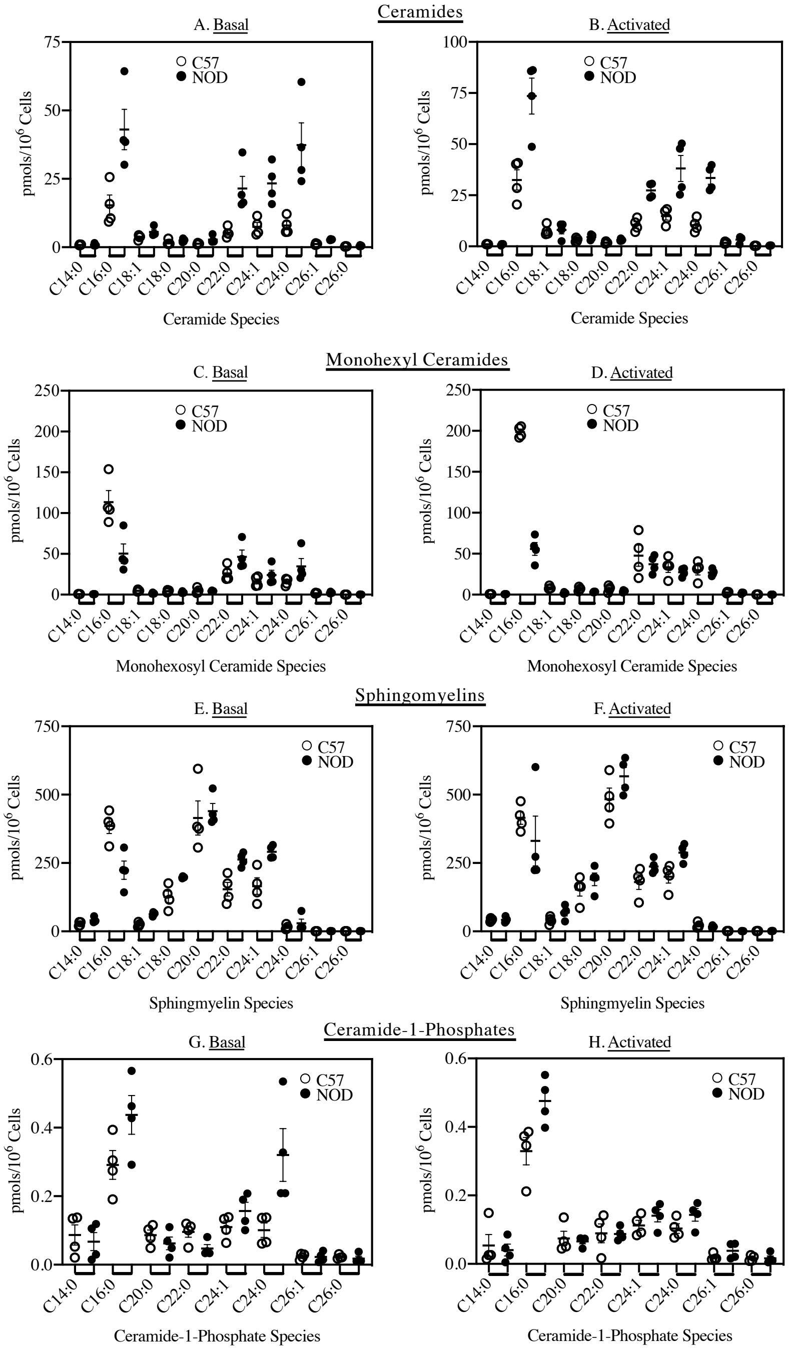

Supplemental Figure 1. Sphingolipids production by MΦ_{NOD} and MΦ_{C57}. Peritoneal macrophages isolated from female NOD (n=4) and age-matched C57BL/J6 from Jackson Laboratories (n=4) were treated with vehicle (DMSO, basal) alone or classically activated with IFNγ+LPS and the cells collected for sphingolipids analyses. The data (mean ± SEMs) are pmol lipid/10^6 cells. (Statistical analyses: Students' t-test. MΦ_{NOD} significantly different from MΦ_{C57}, $^{\dagger}p < 0.05$; $^{c}p < 0.01$; $^{\#}p < 0.005$; $^{¥}p < 0.001$, $^{*}p < 0.0001$.)

Supplemental Figure 2. Select Temporal Eicosanoid Production by $M\Phi_{NOD}$ and $M\Phi_{NOD\text{-}HET}$

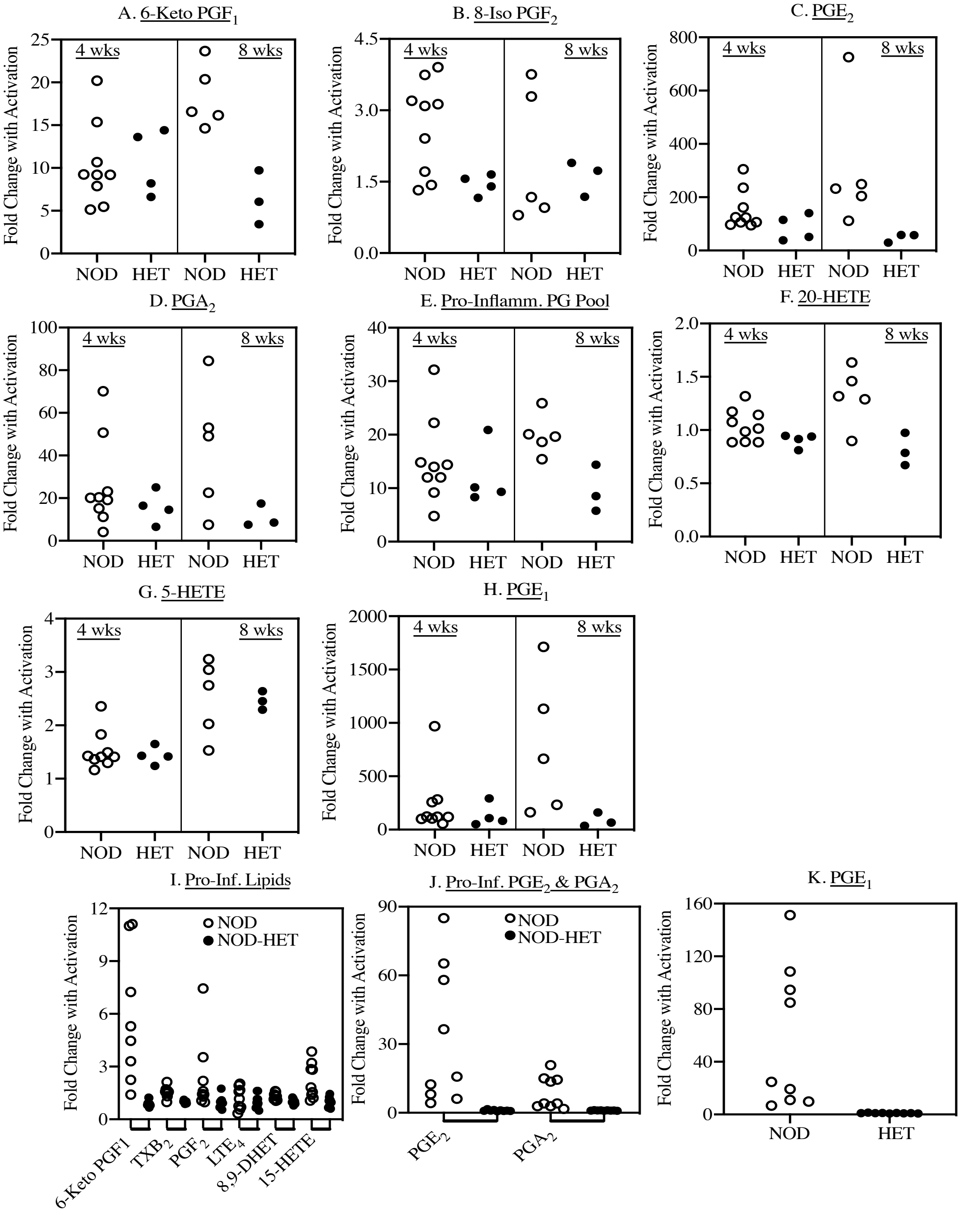

Supplemental Figure 2. Comparison of eicosanoid production by $M\Phi_{NOD}$ and $M\Phi_{NOD\text{-}HET}$. Peritoneal macrophages ($M\Phi$) isolated from female NOD and NOD-HET mice were treated with vehicle control (DMSO) or classically activated with IFNγ+LPS and the media collected for eicosanoid analyses at 16h. The data are means ± SEMs of fold-change with activation, relative to corresponding controls. $M\Phi_{NOD}$ (n = 9 & 5) and $M\Phi_{NOD\text{-}HET}$ (n = 4 & 3) at 4 & 8 weeks, respectively. **A**. 6-Keto $PGF_2\alpha$. **B**. 8-Iso $PGF_2\alpha$. **C**. PGE_2. **D**. PGA_2. **E**. Pro-inflammatory pool. **F**. 20-HETE. **G**. 5-HETE. **H**. PGE_1. **I & J**. Proinflammatory and (**K**) anti-inflammatory PGE_1 at 14 week. (n = 9 in each group.)

Supplemental Figure 3. Blood Glucose Monitoring in NOD Mice

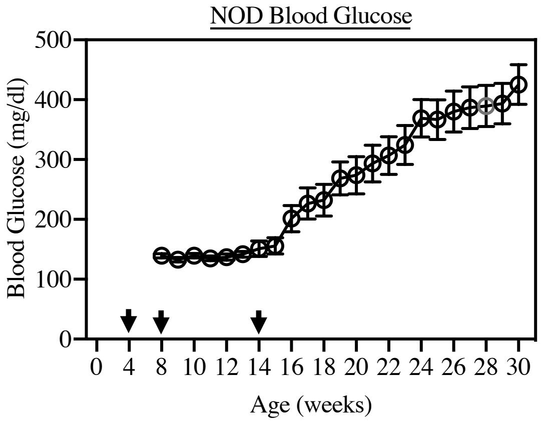

Supplemental Figure 3. Blood glucose monitoring in NOD mice. The data (mean ± SEM) are weekly blood glucose measurements in female NOD mice between 8 and 30 weeks of age. The data are pooled measurements from multiple cohorts (n = 42) utilized in the various studies. The blood glucose values recorded at the onset of diabetes (≥ 275 mg/dl) were carried through till the end of the study for the purpose of generating this figure. Lipidomics analyses were performed with mice at ages (arrows) that were not associated with hyperglycemia.

Supplemental Figure 4. Comparison of Select Plasma Lipids in Diabetic NOD Mice

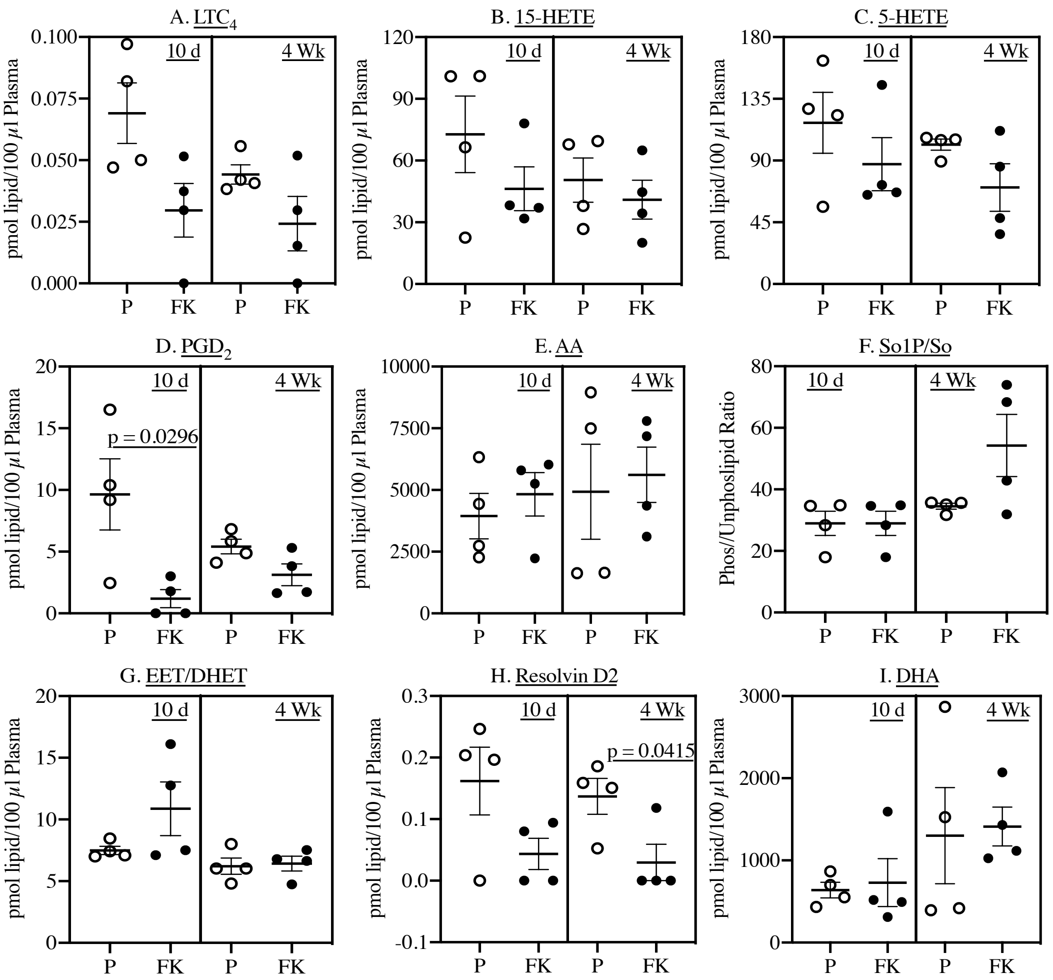

Supplemental Figure 4. Comparison of select plasma lipids at in diabetic NOD mice. NOD mice were treated with PBS-T or with FKGK18, starting at 10 days or 4 weeks of age, and sacrificed at the onset of diabetes. Plasma was prepared from these mice and processed for lipidomics analyses. The data (mean ± SEM) represent pmol of each lipid species in 100 or 50 µL plasma. **A**. LTC_4. **B**. 15-HETE. **C**. 5-HETE. **D**. PGD_2. **E**. AA. **F**. So1P/So. **G**. EET/DHET. **H**. Resolvin D2. **I**. DHA. (Statistical analyses: Students' t-test. N=4/group.)

Supplemental Figure 5. Diabetes Incidence Following Adoptive Transfer of $M\Phi_{NOD}$ and $M\Phi_{NOD\text{-}KO}$

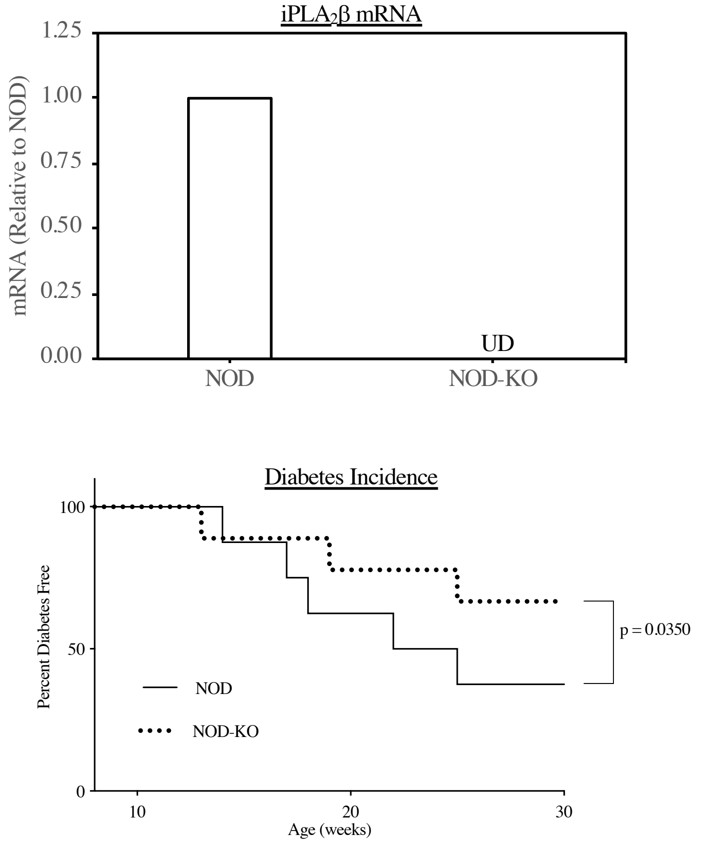

Supplemental Figure 5. Macrophage Adoptive Transfer. Peritoneal macrophages were obtained from 8 week old female NOD and NOD.iPLA$_2\beta^{-/-}$ (NOD-KO). Female NOD mice (at 8 weeks of age) were administered the macrophages (*i.p.*, 2.75 x 10^6) from NOD (n=11) or NOD-KO (n=14). **A**. *iPLA$_2\beta$* mRNA. Macrophage phenotype was verified by RT-qPCR. **B**. Diabetes incidence. Blood glucose was monitored weekly for up to 30 weeks. Two consecutive readings of ≥ 275 mg/dL were recorded as onset of T1D. (Statistical analyses: Mantel-Cox test.) UD, undetected.
